# Nrf2 Drives Epigenetic Reprogramming and Acts as the Master Regulator of KLF4 Expression and Activity in Arsenic‐Induced Transformation

**DOI:** 10.1002/advs.202500221

**Published:** 2025-08-04

**Authors:** Ziwei Wang, Zhuoyue Bi, Jessica Bamrah, Yiran Qiu, Wenxuan Zhang, Bandar Saeed Almutairy, Haoyan Ji, John D. Haley, Chitra Thakur, Fei Chen

**Affiliations:** ^1^ Stony Brook Cancer Center and Department of Pathology Renaissance School of Medicine Stony Brook University Lauterbur Drive Stony Brook NY 11794 USA; ^2^ Department of Pharmaceutical Sciences College of Pharmacy Shaqra University Shaqra 11961 Saudi Arabia; ^3^ Stony Brook University Proteomics Center Renaissance School of Medicine Stony Brook University 110 Nicolls Road Stony Brook NY 11794 USA

**Keywords:** arsenic, carcinogenesis, cell malignancy, KLF4, Nrf2

## Abstract

Emerging evidence suggests that Nrf2 plays a pro‐carcinogenic role in multiple cancers. Previous studies have shown that arsenic‐induced Nrf2 activation drives metabolic reprogramming, leading to the formation of cancer stem‐like cells. Here, we further demonstrate that KLF4, a key pluripotency factor, is a direct transcriptional target of Nrf2 in arsenic‐treated human bronchial epithelial cells (BEAS‐2B). Global chromatin immunoprecipitation sequencing (ChIP‐seq) revealed multiple Nrf2 binding peaks within the *KLF4* locus, overlapping with the enhancer markers H3K4me1 and H3K27Ac. Nrf2 knockout diminished KLF4 expression and reduced enhancer marker enrichment, accompanied by a global decrease in KLF4 genomic occupancy in BEAS‐2B cells. Additionally, arsenic exposure enhances KLF4 binding at genes implicated in oncogenic pathways, including STAT3, SOX2, Nrf2 itself, cell growth regulators, Hedgehog signaling components, and epithelial‐to‐mesenchymal transition (EMT) markers. Notably, we identified a self‐reinforcing feedback loop between Nrf2 and KLF4 that amplifies their oncogenic effects. Finally, our data reveal that Nrf2‐KLF4 co‐occupancy is essential for establishing active enhancer hubs across the genome. Collectively, these findings suggest that Nrf2's oncogenic effects are, in part, mediated through the Nrf2‐dependent amplification of KLF4 expression and function. Targeting both Nrf2 and KLF4 may, therefore, represent a promising therapeutic strategy to eliminate arsenic‐induced cancer stem‐like cells.

## Graphical abstract

Nrf2 isn't just a protector—it's a driver of cancer stemness. The study uncovers how arsenic‐activated Nrf2 directly upregulates KLF4, a key pluripotency factor, fueling oncogenic reprogramming. Through enhancer activation and self‐reinforcing loop, Nrf2 and KLF4 co‐opt gene networks linked to epithelial‐to‐mesenchymal transition and tumor growth.

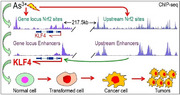

## 1 Introduction

The mechanisms by which environmental and other risk factors contribute to human cancer development remain incompletely understood. Aberrant activation of Nrf2, or somatic mutations in *NFE2L2* (NFE2 like bZIP transcription factor 2), which encodes the cap'n’collar leucine zipper transcription factor Nrf2, are frequently observed in multiple human cancers^[^ ^1^, ^2^ ^]^ Notably, ~25–30% of non‐small cell lung cancers (NSCLCs) exhibit hyperactive Nrf2 due to mutation in *NFE2L2* or its negative regulator, *KEAP1* (Kelch‐like ECH‐associated protein 1).^[^ ^3^, ^4^ ^]^ While Nrf2 is primarily recognized as an oncogenic driver due to its role in upregulating cytoprotective genes in response to oxidative and electrophilic stresses from environmental hazards, some earlier studies have suggested a potential tumor‐suppressive function. In a mouse model of NSCLC, constitutive Nrf2 activation was shown to initiate lung tumor development and drive early progression from hyperplasia to low‐grade tumor, though it did not lead to advanced malignancies.^[^ ^5^ ^]^ However, emerging evidence in human lung cancer suggests that sustained Nrf2 activation promotes metastasis, therapeutical resistance, tumor recurrence, and poor prognosis.^[^ ^6^, ^7^ ^]^ It is believed that Nrf2‐driven heme catabolism may lead to the accumulation of Bach1 (BTB domain and CNC homolog 1), a pro‐metastatic protein.^[^ ^8^ ^]^ Additionally, persistently activated Nrf2 in NSCLCs has been shown to cooperate with CEBPB (CCAAT/enhancer binding protein beta) to remodel enhancers at oncogenic loci typically unregulated by transient Nrf2 activity.^[^ ^9^ ^]^ In pancreatic cancer, Nrf2 also plays a crucial role in sustaining tumor growth by enhancing mRNA translation through the EGFR (epidermal growth factor receptor) and AKT kinase signaling pathways.^[^ ^10^ ^]^

We previously demonstrated that Nrf2 serves as a master regulator of metabolic reprogramming and cancer stem‐like cell (CSC) generation in the human bronchial epithelial cell line BEAS‐2B exposed to environmentally relevant concentrations of arsenic (As^3+^).^[^ ^11^, ^12^ ^]^ Beyond its well‐established role in antioxidant defense, global chromatin immunoprecipitation with sequencing (ChIP‐seq) analysis identified several Nrf2‐regulated genes essential for shifting metabolism from the mitochondrial citric acid (TCA) cycle to glycolysis and glycolytic shunting pathways. This is consistent with recent evidence supporting Nrf2's role in glycolytic metabolism across diverse cell types, including prostate stem progenitor cells,^[^ ^13^ ^]^ human hepatocarcinoma cells,^[^ ^14^ ^]^ breast cancer cells,^[^ ^15^ ^]^ oral squamous cell carcinoma,^[^ ^16^ ^]^ T cells,^[^ ^17^, ^18^ ^]^ cardiomyocytes,^[^ ^19^ ^]^ endothelial cells,^[^ ^20^ ^]^ and others.^[^ ^21^, ^22^, ^23^ ^]^

A distinctive feature of Nrf2‐driven glycolysis in As^3+^‐transformed cells or CSCs is the active diversion of glycolytic intermediates into the hexosamine biosynthetic and serine‐glycine pathways.^[^ ^11^ ^]^ While Nrf2 promotes glycolytic metabolism, recent studies suggest that glycolytic intermediates, such as glyceraldehyde 3‐phosphate, reciprocally activate Nrf2 by modifying KEAP1 through S‐lactoyl modification, leading to its inactivation.^[^ ^24^ ^]^ Given KEAP1's role in targeting Nrf2 for ubiquitination and proteasomal degradation, this modification establishes a mutual amplification loop between Nrf2 activity and glycolytic metabolism.

Glycolysis is well known to support the stemness of stem cells, progenitor cells, cancer cells, and CSCs.^[^ ^25^ ^]^ Certain glycolytic metabolites, including uridine diphosphate N‐acetylglucosamine (UDP‐GlcNAc) and NADPH (nicotinamide adenine dinucleotide phosphate), are essential for activating pluripotency transcription factors. Beyond metabolic regulation, Nrf2 may also directly induce the expression of stemness‐associated genes. Transient As^3+^ exposure significantly enriched Nrf2 occupancy at the genes encoding key pluripotency and stem cell markers, including *MYC*, *SOX2 (*SRY‐Box transcription factor 2), *KLF4* (KLF transcription factor 4), *CD44*, *EGFR*, *BACH1*, *FGF1* (fibroblast growth factor 1), *NGF* (nerve growth factor), and *PDGFB* (platelet‐derived growth factor subunit B), highlighting Nrf2 as a pivotal driver of CSC formation and functional specialization in carcinogenesis.^[^ ^11^, ^26^ ^]^

In this report, we provide new evidence that Nrf2 orchestrates the epigenetic regulation of *KLF4* expression and function. ChIP‐seq analysis identified multiple Nrf2 binding peaks overlapping with key enhancer markers—monomethylation of histone H3 at lysine 4 (H3K4me1) and histone 3 lysine 27 acetylation (H3K27Ac)—within the KLF4 gene loci. Nrf2 knockout reduced H3K4me1 and H3K27Ac levels at the KLF4 gene, along with diminished KLF4 expression and DNA‐binding activity in both As^3+^‐treated and As^3+^‐transformed cells. These findings suggest that targeting Nrf2 or its downstream effector KLF4 could be a novel strategy against As^3+^‐driven carcinogenesis.

## 2 Results

### 2.1 Nrf2 Dependency in As^3+^‐Induced Malignant Transformation

**Figure 1:**
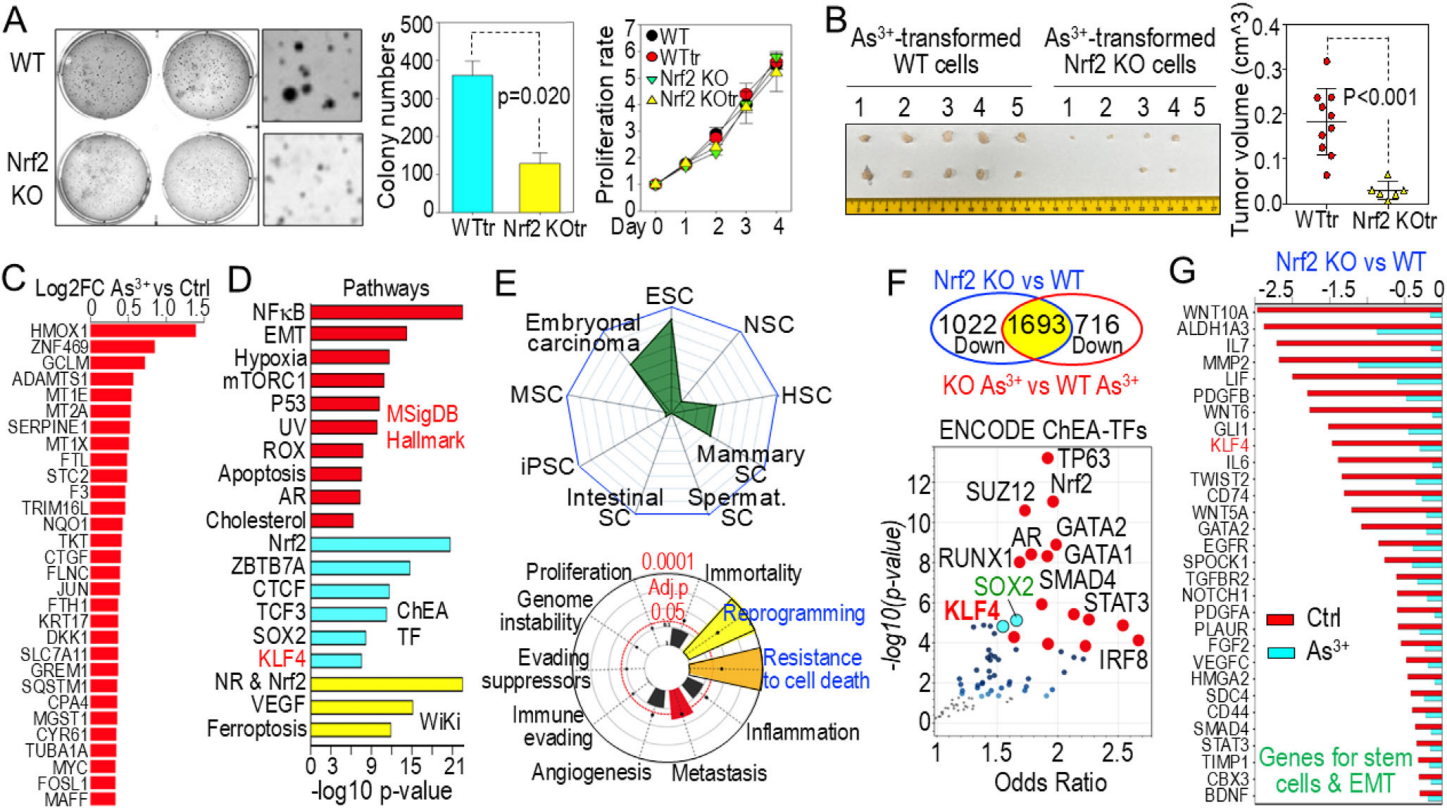
Nrf2 is essential for As^3+^‐induced malignant transformation. A) Anchorage‐independent growth of WT and Nrf2 KO cells treated with 0.25 µm As^3+^ for three months in soft agar. Middle panel quantifies cell colonies (>100 µm in diameter) in soft agar from three independent experiments (n = 3 per group per experiment). Right panel shows the proliferation of As^3+^‐transformed WT and Nrf2 KO cells (n = 6 per group). Data are presented as mean ± SD. Statistical significance was determined using Student's *t*‐test (middle panel) or paired one‐way ANOVA with Bonferroni's multiple comparisons test (right panel). B) Representative images of tumors formed by subcutaneous inoculation of As^3+^‐transformed WT and Nrf2 KO cells in NSG mice (n = 5 per group). The right panel displays quantified *ex vivo* tumor volumes. Data are presented as mean ± SD. Statistical significance was determined using Student's *t*‐test. C) RNA‐seq analysis showing the top 30 most upregulated genes in WT cells after treatment with 2 µm As^3+^ for 12 h. Results are shown as average log2 fold changes (n = 3 per group). D) Enrichr pathway analysis of As^3+^‐induced genes in WT cells. Results are shown as log *p*‐value. E) Profiling of As^3+^‐induced stemness‐related genes using Stemchecker (top) and cancer pathway analysis using Hallmark Enrichment Plot (bottom). ESC: embryonic stem cell; NSC: neural stem cell; HSC: hematopoietic stem cell; iPSC: induced pluripotent stem cell; MSC: mesenchymal stem cell. F) Identification of Nrf2‐dependent genes by comparing differentially expressed genes between Nrf2 KO and WT cells with or without As^3+^ treatment. Bottom panel shows major transcription factor pathways regulating these Nrf2‐dependent genes. G) Reduced expression of stemness‐ and EMT‐ related genes in Nrf2 KO cells. RNA‐seq data were obtained from three independent biological replicates per group. Results are presented as average log2 fold changes (Nrf2 KO versus WT) (n = 3 per group).

We previously demonstrated that Nrf2 plays an essential role in As^3+^‐induced metabolic reprogramming and CSC formation.^[^ ^11^, ^26^ ^]^ To further investigate this, we treated both wild‐type (WT) and CRISPR‐edited Nrf2 knockout (KO) BEAS‐2B cells with 0.25 µm As^3+^ for 3 months to assess the impact of Nrf2 on malignant transformation. While both WT and Nrf2 KO cells exhibited characteristics of malignant transformation, as evidenced by anchorage‐independent growth in soft agar, the extent of transformation differed markedly (**Figure** **1**A, left panel). WT cells formed numerous large colonies, whereas Nrf2 KO cells primarily generated small colonies and individual resting cells. The cell proliferation assay showed no significant difference in in vitro proliferation rates between WT and Nrf2 KO cells, regardless of As^3+^ exposure (Figure 1A, right panel). This suggests that while Nrf2 knockout attenuates malignant transformation, it does not impact overall cell growth. To further evaluate the oncogenic potential of Nrf2, we conducted a mouse xenograft assay. In previous studies, we demonstrated that non‐transformed WT and Nrf2 KO cells are unable to form tumors in mouse xenograft tests,^[^ ^27^ ^]^ consistent with earlier reports.^[^ ^28^, ^29^ ^]^ By week 8, subcutaneous injection of As^3+^‐transformed WT cells resulted in large tumors, whereas tumors derived from As^3+^‐transformed Nrf2 KO cells were significantly smaller (Figure 1B), reinforcing the pro‐malignant role of Nrf2 in As^3+^‐induced carcinogenesis. Moreover, given the lack of difference in cell proliferation between WT and Nrf2 KO cells, these findings suggest that the tumorigenic effects of Nrf2 are not simply a byproduct of altered cell viability following its knockout. Rather, they highlight a fundamental role of Nrf2 in driving stemness and malignancy.

To uncover the initial molecular events through which Nrf2 facilitates As^3+^‐induced malignant transformation, we performed RNA sequencing (RNA‐seq) on WT and Nrf2 KO cells, either untreated or treated with 2 µm As^3+^ for 12 h. In WT cells, As^3+^ exposure led to the induction of 923 genes with a shrunken Log2 fold change (FC) ≥ 0.1. Among the top 30 upregulated genes were several well‐known Nrf2 target genes, including *HMOX1, NQO1, SQSTM1*, and members of the metal‐induced metallothionein family (*MT1E*, *MT2A*, and *MT1X*) (Figure 1C). Pathway enrichment analysis identified key cancer‐related pathways such as NF‐κB signaling, EMT, hypoxia, and mTORC1 as significantly enriched. Notably, transcriptional programs associated with stemness, including *KLF4, SOX2*, and *TCF3*, were consistently overrepresented in analyses using ChIP‐X of ChEA transcription factors (TFs) and other databases (Figure 1D). Further support this observation, StemCheaker analysis revealed significant enrichment of genes linked to embryonal carcinoma, embryonic stem cells (ESCs), hematopoietic stem cells (HSCs), and mammary stem cells. Integrated Cancer Hallmark Gene Set analysis also highlighted significant enrichment of genes involved in metabolic reprogramming, resistance to cell death, and metastasis (Figure 1E).

To identify genes induced by As^3+^ in an Nrf2‐dependent manner, we focused on those that were both upregulated by As^3+^ and require Nrf2 for expression. Specifically, we identified 1693 genes whose expression was significantly reduced in Nrf2 KO cells compared to WT cells under As^3+^ treatment. Analysis of ENCODE and ChEA Consensus TFs from ChIP‐X revealed that these genes are predominantly regulated by transcription factors involved in cell growth, stress response, and CSC development, including TP63, Nrf2, SOX2, KLF4, GATA2, SMAD4, IRF8, and STAT3 (Figure 1F). As shown in Figure 1G, Nrf2 knockout markedly downregulated the expression of key stemness‐ and EMT‐ associated genes, including WNT family members, *ALDH1A3, IL7, MMP2, LIF, KLF4, NOTCH1, SPOCK1*, and *CD44*. These findings underscore the pivotal role of Nrf2 in As^3+^‐induced malignant transformation and the potential emergence of CSCs.

### 2.2 Clinical Relevance of Nrf2 in Human Cancers

**Figure 2:**
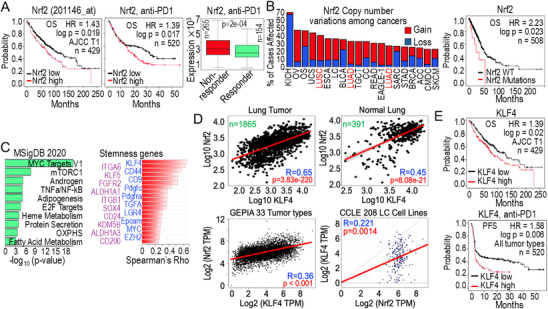
Oncogenic properties of Nrf2 in human cancers. A) High levels of Nrf2 predict poorer overall survival in lung cancer patients (left, n = 429) and in cancer patients undergoing anti‐PD1 immune therapy (middle, n = 520). The right panel shows increased expression of the Nrf2 gene in non‐responder group of NSCLC patients subjected to anti‐PD1 immune therapy (https://www.rocplot.com/immune). Statistical significance in right panel was determined using Mann‐Whitney test. B) Copy number variations of the Nrf2 gene across different human tumors (left). The right panel shows the association between Nrf2 gene mutation and poorer overall survival in lung cancer patients. C) Molecular Signature Cancer Hallmark pathway analysis of 604 genes positively correlated with Nrf2 (Spearman's Rho ≥ 0.5) in 1865 human lung tumors. (Right) The Top 20 stemness‐associated genes enriched in MYC signaling pathways were among those most highly correlated with Nrf2 expression. D) *Nrf2 (NFE2L2)* is the top *KLF4*‐correlated genes in human lung cancer and one of the most highly correlated genes with *KLF4* in normal human lung tissue. Lower left: Positive correlation of Nrf2 and KLF4 expression across 33 different tumor types in the GEPIA database. Lower right: Positive correlation of Nrf2 and KLF4 expression in 208 lung cancer cell lines from the CCLE. E) High *KLF4* expression predicts poor overall survival in lung cancer patients and reduced PFS in patients undergoing anti‐PD1 ICB therapy.

To assess the clinical relevance of our findings, we first evaluated the prognostic significance of Nrf2 expression in a large lung cancer cohort using gene expression profiles measured by two distinct Nrf2 probes (201146_at and 1567013_at), based on publicly available datasets from the Kaplan‐Meier Plotter platform. Both probes revealed a strong association between elevated Nrf2 expression and poorer survival outcomes in lung cancer patients at the AJCC T1 and T2 stage, regardless of smoking status or histological subtypes (**Figure** **2**A, left, and data not shown). Notably, high Nrf2 expression was also correlated with worse survival outcomes in cancer patients undergoing immune checkpoint inhibitor therapies, including anti‐PD1 and anti‐CTLA‐4 treatments, irrespective of cancer types (Figure 2A, middle). Among 154 responders and 265 non‐responders to anti‐PD1 therapy in NSCLC, Nrf2 expression was significantly higher in the non‐responders (Figure 2A, right), suggesting a potential role of Nrf2 in mediating resistance to immunotherapy.

Given that Nrf2 functions as a transcription factor, its activity, rather than its mere expression, may play a critical role in cancer progression. To explore this, we examined the correlation between Nrf2 gene mutations and overall survival in lung cancer patients. Analysis of data from the NCI Genomic Data Commons (GDC) Data Portal revealed that multiple cancer types, particularly lung squamous carcinoma (LUSC) and lung adenocarcinoma (LUAD), frequently exhibited copy number variations (CNV) gains in the Nrf2 gene, which were strongly associated with poorer overall survival (Figure 2B). The oncogenic role of Nrf2 is further supported by the genes that correlated with its expression in lung cancer. In a cohort of 1865 human lung tumors, we identified 604 genes positively correlated with Nrf2 expression (Spearmen's coefficient (Rho) ≥ 0.5, Table S1, Supporting Information). Many of these genes are MYC target genes or are involved in key signaling pathways, including mTORC1, androgen, and NF‐κB signaling (Figure 2C, left). Notably, several stemness‐associated genes, such as *KLF4*, *CD44*, *ITGA6*, *CD9*, *MYC, ALDH1A1, ALDH1A3*, and *EPCAM*, were among the most highly correlated with Nrf2 expressions (Figure 2C, right). Among them, KLF4 emerged as one of the strongest Nrf2‐associated genes in both lung tumors and normal lung tissues (Figure 2D). This correlation is significantly stronger in lung tumors (Spearman's Rho = 0.65, *p* = 3.83e‐220) compared to normal lung tissues (Rho = 0.45, *p* = 8.80e‐21), possibly reflecting the paradoxical roles of these two factors in lung tumors versus normal lung tissues. In the GEPIA (Gene Expression Profiling Interactive Analysis) dataset, which contains 33 different tumor types, Nrf2 and KLF4 show a notable positive correlation. A similar correlation is also observed among 208 lung cancer cell lines derived from various histological subtypes of lung tumors in the Cancer Cell Line Encyclopedia (CCLE). Furthermore, higher KLF4 expression is associated with poorer overall survival (OS) in lung cancer patients, as well as reduced progression‐free survival (PFS) in patients undergoing anti‐PD1 (Immune‐checkpoint inhibitor) ICB therapy (Figure 2E), underscoring its prognostic significance. These findings are consistent with Figure 1D–G, which demonstrate Nrf2‐dependent KLF4 expression and signaling in As^3+^‐induced transformed cells.

### 2.3 KLF4 as a Transcriptional Target of Nrf2 and its Cooperation with Enhancers

**Figure 3:**
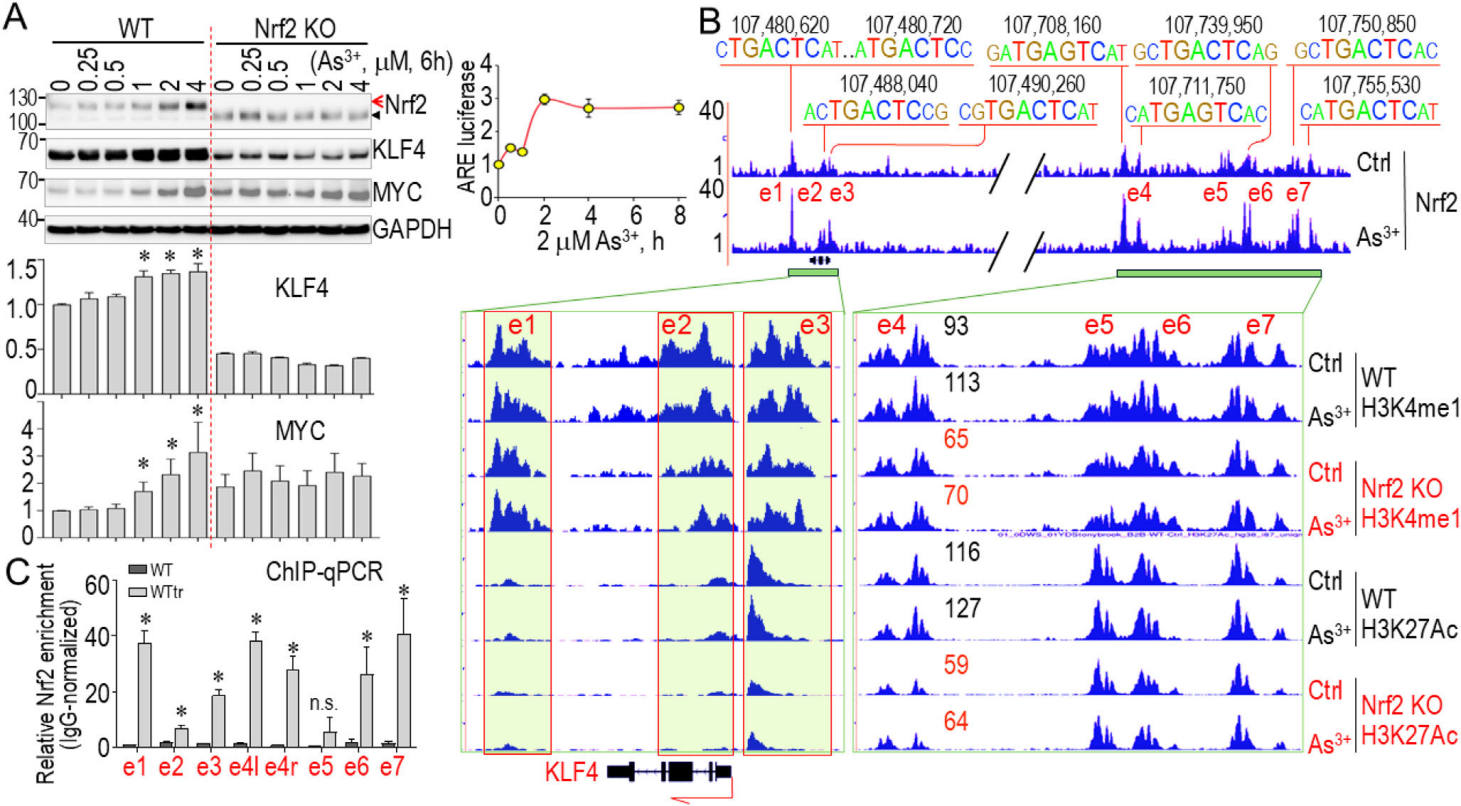
KLF4 is a transcriptional target of Nrf2. A) WT and Nrf2 KO cells treated with the indicated concentrations of As^3+^ for 6 h followed by Western blotting analysis of the indicated proteins. Quantitative results of KLF4 and MYC are shown below. Data are presented as mean ± SD (n = 3). *p < 0.05 versus control by one‐way ANOVA with Bonferroni's multiple comparisons test. (Right) ARE‐Luciferase Reporter Assay for Nrf2 Activation. ARE‐inducible luciferase reporter assay showing Nrf2 activation over 0–8 h of treatment with 2 µm As^3+^. Data are presented as mean ± SD (n = 9). B) ChIP‐seq analysis showing As^3+^‐induced Nrf2 binding and enrichment of enhancer markers H3K4me1 and H3K27Ac at the KLF4 gene locus. ARE elements and their corresponding genomic positions are shown at the top of the panel. Potential enhancers are designated as e1 to e7. The numbers in the bottom right panel indicate the relative degree of enhancer marker enrichment in each group. C) ChIP‐qPCR analysis showing Nrf2 occupancy at the identified ARE elements on the KLF4 locus in transformed WT cells treated with 1 µm As^3+^ for 6 h (WTtr). Data are presented as mean ± SD (n = 9). *p < 0.05 versus WT, determined by Student's *t*‐test.

The strong correlation between Nrf2 and KLF4 in human lung tumors suggests that Nrf2 directly regulates KLF4 expression. In WT cells, transient As^3+^ treatment activates Nrf2 and leads to a dose‐dependent increase in KLF4 and MYC protein levels, two oncogenic transcription factors associated with stemness and CSCs (**Figure** **3**A; Figure S1, Supporting Information). This induction is significantly diminished in Nrf2 KO cells (Figure 3A), reinforcing the role of Nrf2 in driving KLF4 expression. Nrf2 activation by As^3+^ in WT cells was further confirmed using an antioxidant response element (ARE)‐based Nrf2‐dependent luciferase activity assay (Figure 3A, right; Figure S2, Supporting Information). ChIP‐seq analysis revealed robust Nrf2 binding at multiple sites across the KLF4 gene locus following As^3+^ treatment, including regions within the gene body as well as upstream and downstream regulatory elements (Figure 3B). Notably, these Nrf2‐bound regions, designated enhancers e1 through e7, had not been previously characterized and were found to harbor one or two consecutive ARE motifs (Figure 3B). A defining feature of these Nrf2 binding clusters is their colocalization with enhancer‐associated histone marks H3K4me1 and H3K27ac, which were significantly reduced in Nrf2 KO cells, indicating that Nrf2 is essential for maintaining active enhancer states at the KLF4 locus. On a broader genomic scale, our ChIP‐seq data indicate a general tendency for Nrf2 to colocalize with enhancer markers, implying that Nrf2 may coordinate with other epigenic regulators to establish a permissive chromatin environment for the transcriptional activation of *KLF4* and other oncogenic genes. Additional Nrf2 ChIP‐qPCR analysis in As^3+^‐transformed WT cells confirmed enhanced Nrf2 binding in these Nrf2 peaks identified in ChIP‐seq in As^3+^‐treated cells, except for e5, which exhibited high variability (Figure 3C). Collectively, these findings underscore Nrf2 as a key regulator of *KLF4* expression and enhancer activity, highlighting its broader role in shaping the epigenetic landscape to promote stemness and oncogenic transformation.

### 2.4 Nrf2 Regulation of KLF4 in As^3+^‐Transformed Human Induced Pluripotent Stem Cells (iPSCs)

**Figure 4:**
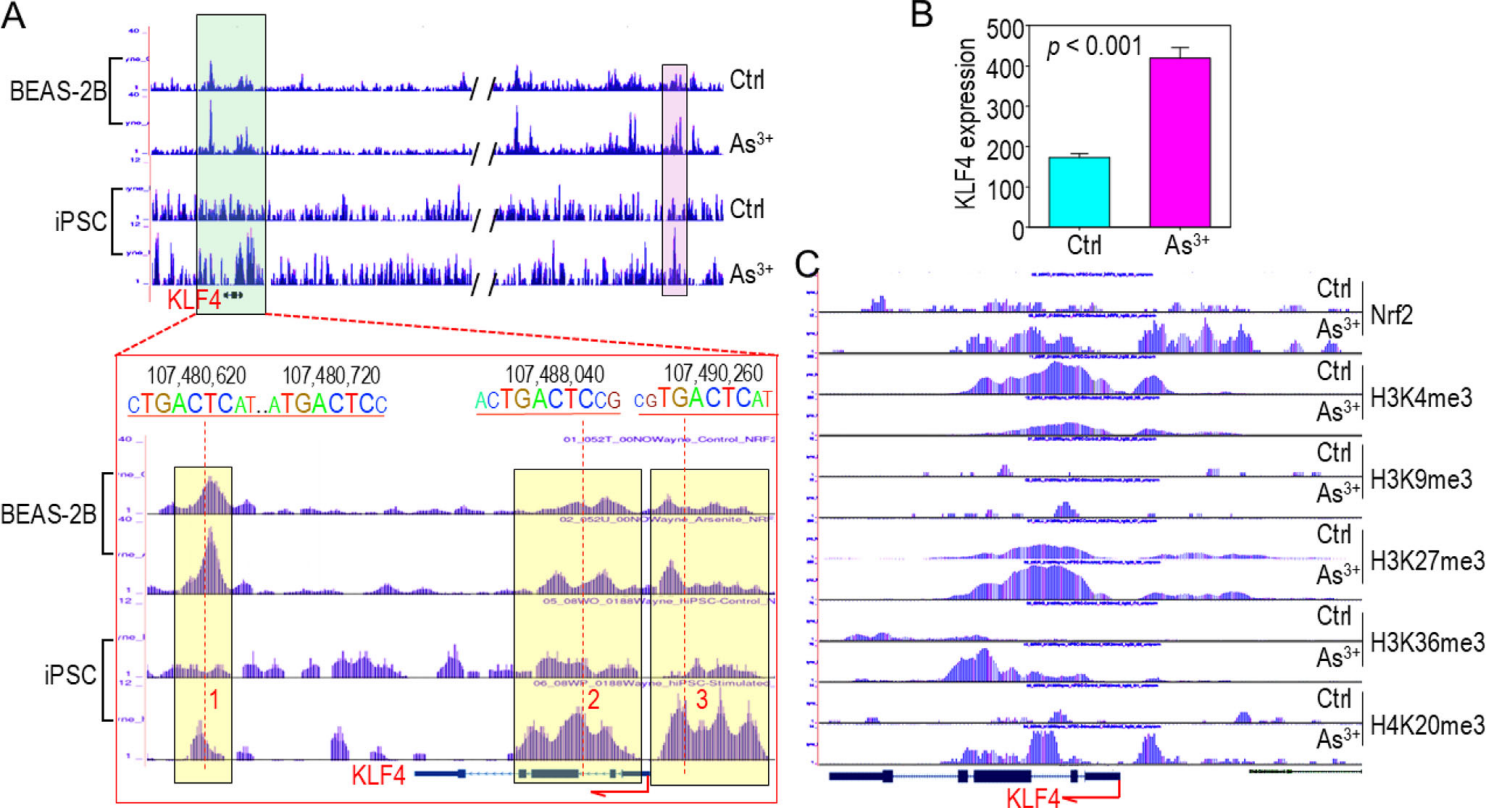
Nrf2 regulates KLF4 expression in As^3+^‐transformed human iPSCs. A) ChIP‐seq comparison of Nrf2 binding profile at the KLF4 gene locus between BEAS‐2B cells and iPSCs. Shared Nrf2‐binding peaks between the two cell types were highlighted. The lower panel illustrates conserved Nrf2‐binding elements within the KLF4 gene. B) RNA‐seq analysis showing *KLF4* induction by As^3+^ in iPSCs. Data are presented as mean ± SD (n = 3, based on count values). *p < 0.05 versus WT, determined by Student's *t*‐test. C) Chromatin methylation status at the KLF4 gene locus, determined by ChIP‐seq using the indicated antibodies in control and As^3+^‐transformed iPSCs.

To investigate whether Nrf2 regulates KLF4 expression in cell types beyond BEAS‐2B, we analyzed our ChIP‐seq data from control and 0.25 µm As^3+^‐transformed iPSCs. These iPSCs were originally generated via Sendai virus‐based transduction of reprogramming factors in human nasal epithelial cells.^[^ ^30^, ^31^ ^]^ We previously demonstrated that chronic exposure to As^3^⁺ for 3 months induces tumor formation in these iPSCs, with tumors exhibiting histological features of both lung squamous cell carcinoma and mucin‐producing adenocarcinoma. ChIP‐seq analysis revealed a marked increase in Nrf2 binding within the KLF4 gene body and downstream regulatory regions following As^3+^ treatment (**Figure** **4**A). Several of these Nrf2 binding peaks overlapped with those observed in As^3+^‐treated BEAS‐2B cells, including peaks 1, 2, and 3. However, subtle differences were noted, such as shifts in peak apexes and the emergence of new sub‐peaks, which may reflect the involvement of distinct Nrf2 co‐factors and/or protein interactors in iPSCs.

RNA‐seq analysis confirmed that As^3+^ exposure significantly upregulated KLF4 expression in iPSCs (Figure 4B). Although direct validation of Nrf2 dependency in iPSCs is limited due to technical challenges in silencing or knocking out Nrf2, the increased Nrf2 occupancy likely promotes transcriptional elongation and activation of KLF4. This may be mediated by enhanced deposition of the elongation‐associated histone mark H3K36me3, while simultaneously mitigating the loss of H3K4me3 and the gain of repressive marks H3K27me3 and H4K20me3 at the KLF4 locus (Figure 4C). These results support a role for Nrf2 in regulating KLF4 expression in As^3^⁺‐transformed iPSCs via chromatin‐level mechanisms.

### 2.5 Active Transcription of KLF4 in Transformed WT Cells

**Figure 5:**
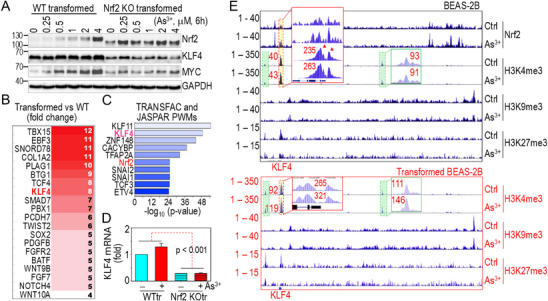
Elevated potential of KLF4 expression in transformed WT cells. A) Western blotting analysis of Nrf2 activation and expression of KLF4 and MYC in transformed WT and Nrf2 KO cells treated with the indicated concentration of As^3+^. B) Expression levels of genes associated with CSCs in transformed WT cells relative to non‐transformed WT cells. RNA‐seq data were obtained from three independent biological replicates per group. Gene expressions are shown as average log2 fold changes (n = 3 per group). C) Transcription factor pathway assay of upregulated genes in transformed WT cells. D) Semi‐quantitative RT‐PCR showing *KLF4* mRNA expression in control and As^3+^‐treated transformed WT (WTtr) and transformed Nrf2 KO (Nrf2 KOtr) cells (n = 6). Statistical significance was determined by paired Student's *t*‐test. E) ChIP‐seq analysis showing histone methylation profiles of the indicated markers at the KLF4 gene locus in non‐transformed (upper panel) and transformed BEAS‐2B cells (bottom panel). Nrf2 ChIP‐seq spectra from control and As^3+^‐treated non‐transformed BEAS‐2B cells are shown at the top of the panel as positional references for the indicated histone methylation markers. The H3K4me3 peaks at the TSS of KLF4 (highlighted in yellow) and up‐ and down‐ stream H3K4me3 peaks (highlighted in green) are indicated.

Similar to non‐transformed cells, transformed WT cells displayed a dose‐dependent induction of KLF4 and MYC proteins in response to As^3+^ (**Figure** **5**A; Figure S3, Supporting Information). However, in transformed Nrf2 KO cells, this As^3+^‐induced upregulation of KLF4 and MYC was significantly reduced. Notably, this pattern differs from that observed in non‐transformed Nrf2 KO cells, where KLF4 expression was diminished, but MYC expression remained largely unchanged (Figures 3A and 5A). Compared to their non‐transformed counterparts, As^3+^‐induced transformed WT cells exhibited significantly higher expression of stemness‐associated genes, including T‐box (TBX) family members, *KLF4*, *TCF4* (Transcription Factor 4), *SOX2*, and several WNT family genes (Figure 5B). A transcription factor pathway analysis of 4008 protein‐coding genes (with an adjusted fold change of ≥ 3 in transformed versus non‐transformed WT cells) identified KLF11 and KLF4 as the top transcription factors regulating these genes, followed by ZNF148, CACYBP, TFAP2A, and Nrf2 (Figure 5C). Furthermore, *KLF4* mRNA levels were significantly lower in transformed Nrf2 KO cells compared to transformed WT cells (Figure 5D), reinforcing the dependency of KLF4 expression on Nrf2 in transformed cells.

It is well‐established that H3K4me3 enrichment and peak breadth at the transcription start site (TSS) or promoter region correlate with gene transcriptional activity. Genes with higher or broader H3K4me3 peaks tend to be more actively transcribed.^[^ ^32^, ^33^, ^34^, ^35^ ^]^ At the KLF4 gene locus, two broad H3K4me3 peaks were identified, which aligned with Nrf2 binding sites—one located 510 bp upstream of the TSS and another spanning from exon 1 to exon 3, peaking at the +770 bp region (highlighted by red triangles; Figure 5E, upper). Notably, transformed WT cells exhibited increased H3K4me3 enrichment alongside a reduction in transcriptional repression markers H3K9me3 and H3K27me3 at the KLF4 gene locus, suggesting a chromatin state favoring active transcription. In addition to the H3K4me3 peaks near the TSS, two intergenic H3K4me3 peaks were identified—one located 148 kb upstream and another 24 kb downstream of the KLF4 gene (highlighted in green rectangles). Both of these intergenic H3K4me3 peaks were enhanced in As^3+^‐transformed cells (Figure 5E, bottom), providing additional evidence that KLF4 transcription is actively upregulated during malignant transformation.

### 2.6 Nrf2 Participates in Enhancer Remodeling

**Figure 6:**
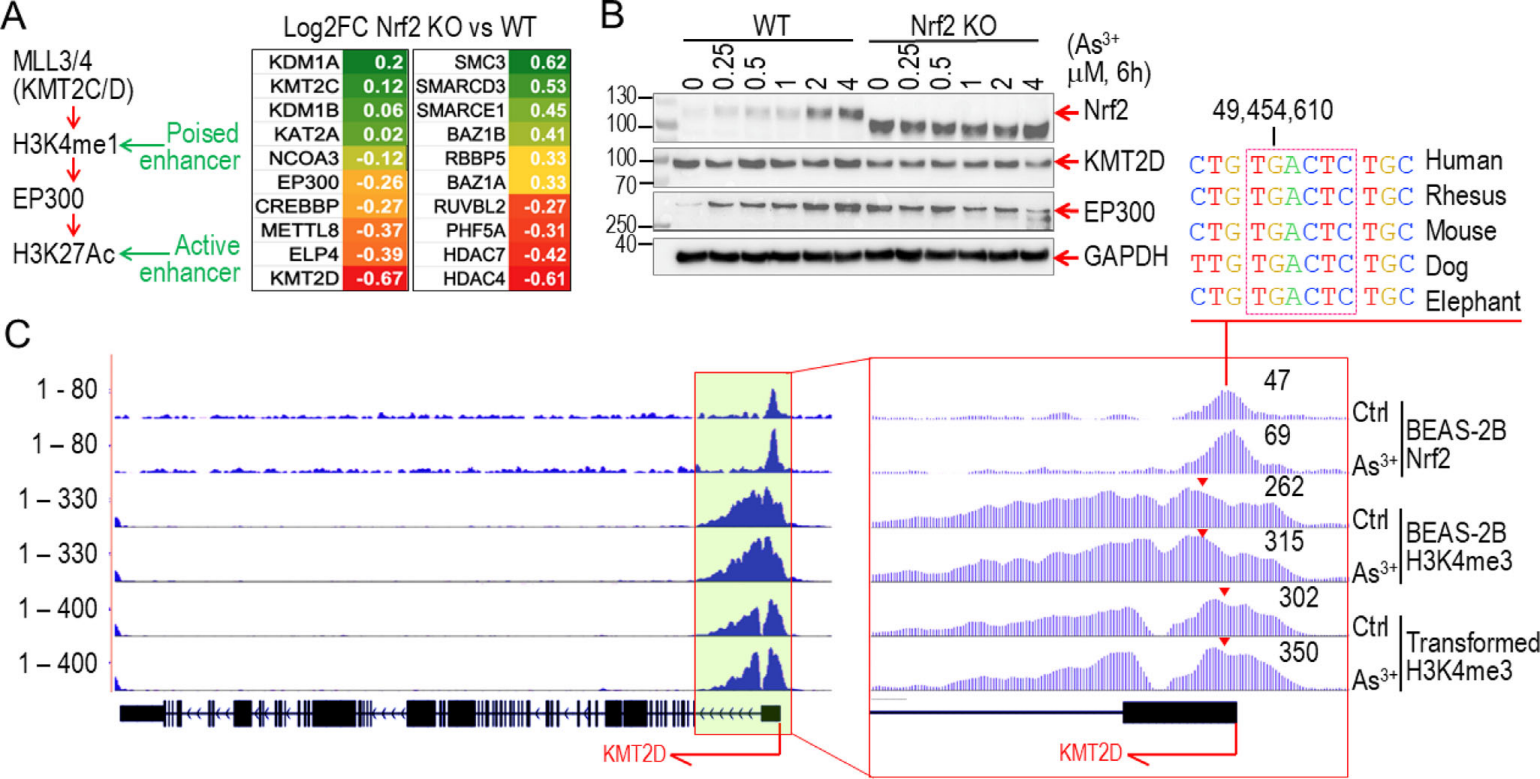
Nrf2 contributes to the establishment of enhancers. A) Schematic diagram illustrating the establishment of poised and active enhancers. The right panel indicates the expression levels of enhancer‐related genes in Nrf2 KO cells relative to WT cells. RNA‐seq data were obtained from three independent biological replicates per group. Expression data are shown as log2 fold changes. B) Western blot analysis showing that knocking out Nrf2 reduces the expression of KMT2D and EP300. C) ChIP‐seq analysis demonstrating that As^3+^ enhances the enrichment of Nrf2 and H3K4me3 at TSS of KMT2D in the indicated cells. The conserved Nrf2‐binding ARE element is indicated at the top of the panel.

The abnormal activation of enhancers, particularly those linked to oncogenic and stemness‐related genes, can establish self‐sustaining transcriptional circuits in transformed or cancerous cells. Although ChIP‐seq analysis revealed no significant differences in the overall levels of H3K4me1 and H3K27Ac between WT and Nrf2 KO cells, the loss of Nrf2 led to a marked reduction of these markers specifically at the KLF4 gene locus (Figure 3B). This suggests that Nrf2 plays a crucial role in the remodeling of *KLF4* enhancers. Extensive research has shown that H3K4me1, catalyzed by KMT2C (lysine methyltransferase 2C) or KMT2D, marks the poised state of enhancers, while the recruitment of the acetyltransferase EP300 by H3K4me1 induces H3K27Ac, converting enhancers into their fully active state. To investigate how Nrf2 depletion diminishes H3K4me1 and H3K27Ac levels at the *KLF4* locus, we examined the impact of Nrf2 knockout on the expression of key enhancer regulators, including methyltransferases responsible for H3K4me1, acetyltransferases mediating H3K27Ac, and genes associated with enhancer complex such as Compass, Cohesin, and the BAF complex. As shown in **Figure** **6**A, the expression of KMT2D, a key methyltransferase for H3K4me1, was significantly reduced in Nrf2 KO cells. Additionally, acetyltransferases involved in H3K27Ac deposition, including EP300, CREBBP (CREB binding protein), and ELP4 (elongator acetyltransferase complex subunit 4), exhibited slight reductions following Nrf2 depletion. This was further corroborated by decreased protein levels of KMT2D and EP300 in Nrf2 KO cells (Figure 6B; Figure S4, Supporting Information), underscoring the essential role of Nrf2 in the expression of these key enhancer regulators. This finding aligns with the observed enrichment of Nrf2 and H3K4me3 at the promoter region of the KMT2D gene in As^3+^‐treated and transformed cells. Notably, a conserved ARE site is present at the peak of Nrf2 enrichment (Figure 6C), providing clear evidence that *KMT2D* is an Nrf2‐target gene involved in enhancer activation at the KLF4 gene locus and other genomic regions.

### 2.7 Ablation of Nrf2 Diminishes Genome‐Wide KLF4 Binding in Response to As^3+^

**Figure 7:**
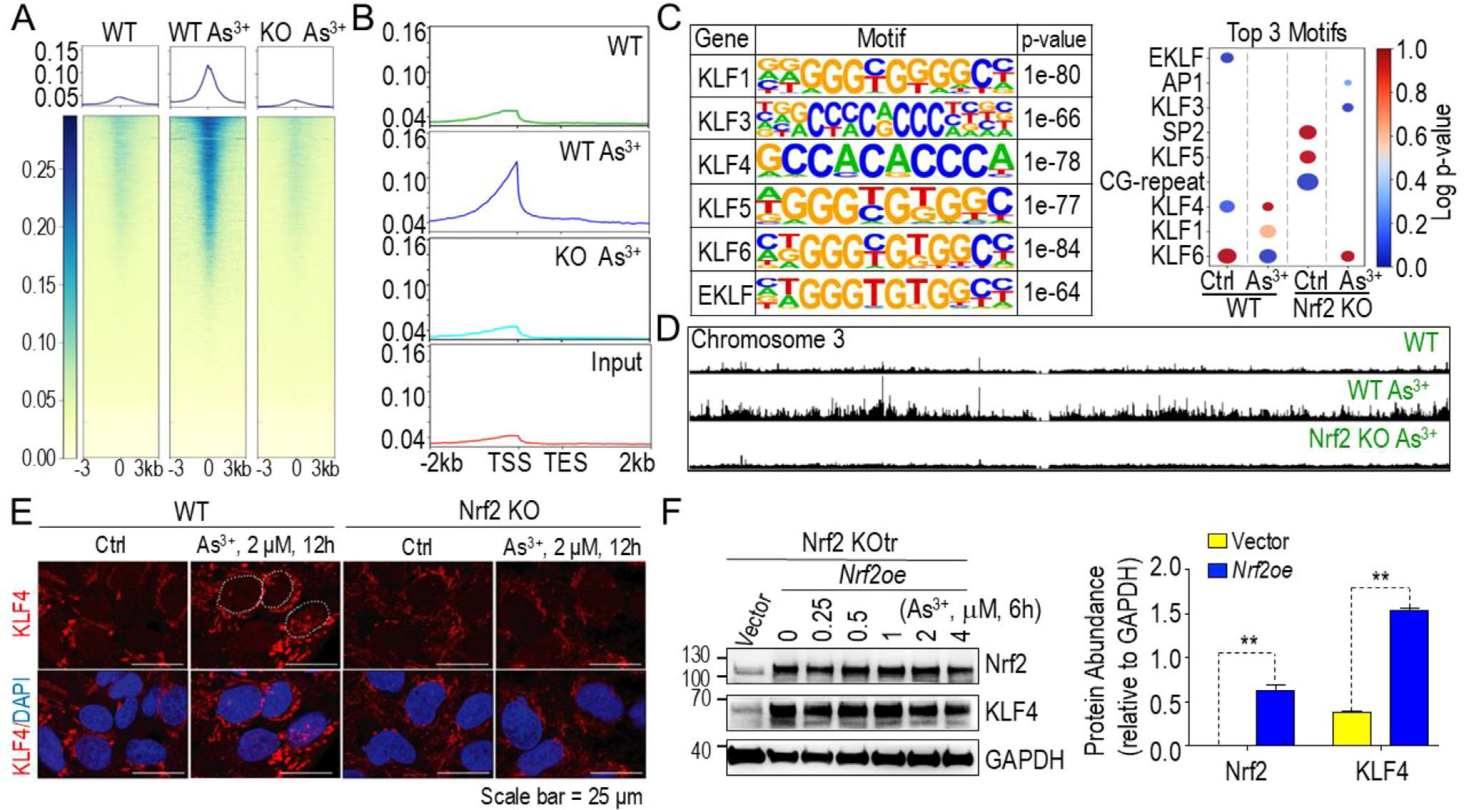
Knocking out Nrf2 blocks As^3+^‐induced genome‐wide KLF4 binding. A) ChIP‐seq heatmaps showing KLF4 binding in control and As^3+^‐treated WT and Nrf2 KO cells. B) Distribution of KLF4 enrichment on gene loci in the indicated cells. C) Top‐enriched KLF elements in the indicated cells with or without As^3+^ treatment. D) Screenshot of KLF4 binding across the entire chromosome 3 in the indicated cells as determined by ChIP‐seq. E) Photomicrographs illustrating the nuclear translocation of KLF4 in WT cells treated with 2 µm As^3+^ for 12 h. Scale bar = 25 µm. F) Western blot analysis of Nrf2, KLF4, and GAPDH in transformed Nrf2 KO (Nrf2 KOtr) cells and Nrf2‐reexpressing (Nrf2oe) Nrf2 KOtr cells following treatment with the indicated concentrations of As^3+^ for 6 h. The right panel presents the relative quantification of Nrf2 and KLF4 protein levels in Nrf2 KOtr cells with or without Nrf2 re‐expression. Data are presented as mean ± SD (n = 6 per group). **p < 0.01 versus Nrf2 KOtr cells transfected with control vector, determined by Student's *t*‐test.

To further examine the dependency of KLF4 activation on Nrf2 in response to As^3+^, we performed global ChIP‐seq analysis of KLF4 in both WT and Nrf2 KO cells. Consistent with the observed upregulation of KLF4 mRNA and protein, transient As^3+^ exposure triggered a robust genome‐wide enrichment of KLF4 binding in WT cells. However, this enrichment was substantially reduced in Nrf2 KO cells (**Figure** **7**A). In WT cells treated with As^3+^, KLF4 binding peaks were predominantly located near TSS, with additional binding observed in upstream and downstream regulatory regions (Figure 7B). HOMER motif analysis identified six significantly enriched KLF family motifs, including KLF1, KLF3, KLF4, KLF5, KLF6, and EKLF, across all experimental groups. Notably, in As^3+^‐treated WT cells, the most enriched motifs at KLF4 peaks were KLF4, KLF1, and KLF6 (Figure 7C). In contrast, the top motifs in untreated Nrf2 KO cells were SP2 (Sp2 transcription factor), KLF5, and CG‐repeat binding protein, while those in As^3+^‐treated Nrf2 KO cells were AP1, KLF3, and KLF6, highlighting distinct motif landscapes between WT and KO contexts. Under basal conditions, KLF4 binding was minimal in WT cells, with only a few weak peaks observed genome‐wide, as exemplified by chromosome 3 (Figure 7D). Following As^3+^ treatment, multiple strong KLF4 binding clusters emerged in WT cells, whereas these peaks were nearly absent in Nrf2 KO cells, emphasizing the essential role of Nrf2 in facilitating KLF4 chromatin engagement. This notion was further supported by KLF4 immunofluorescent staining. In WT cells, As^3+^ treatment led to increased cytoplasmic and nuclear accumulation of KLF4. In contrast, this response was largely absent in Nrf2 KO cells (Figure 7E). To confirm the role of Nrf2 in regulating KLF4 expression, we reintroduced Nrf2 into As^3+^‐transformed Nrf2 KO cells using a pCDH‐NRF2 expression vector. Cells were subsequently treated with increasing concentrations of As^3+^ (0.25–4 µm) for 6 h. As expected, Nrf2 expression was already significantly elevated following vector‐mediated reconstitution and appeared to reach a saturation point, with no further dose‐dependent increase upon As^3+^ treatment. In contrast, KLF4 expression was not only restored but also increased in a dose‐dependent manner (0–1 µm; Figure 7F). Quantitative analysis revealed a strong positive correlation between Nrf2 and KLF4 expression in transformed Nrf2 KO cells following Nrf2 re‐expression and As^3+^ exposure, with a coefficient of determination (R^2^) value of 0.91 (*p* = 0.003). These results underscore the essential role of Nrf2 in mediating KLF4 activation in response to As^3+^ exposure.

### 2.8 Unique Characteristics of KLF4‐Regulated Genes

**Figure 8:**
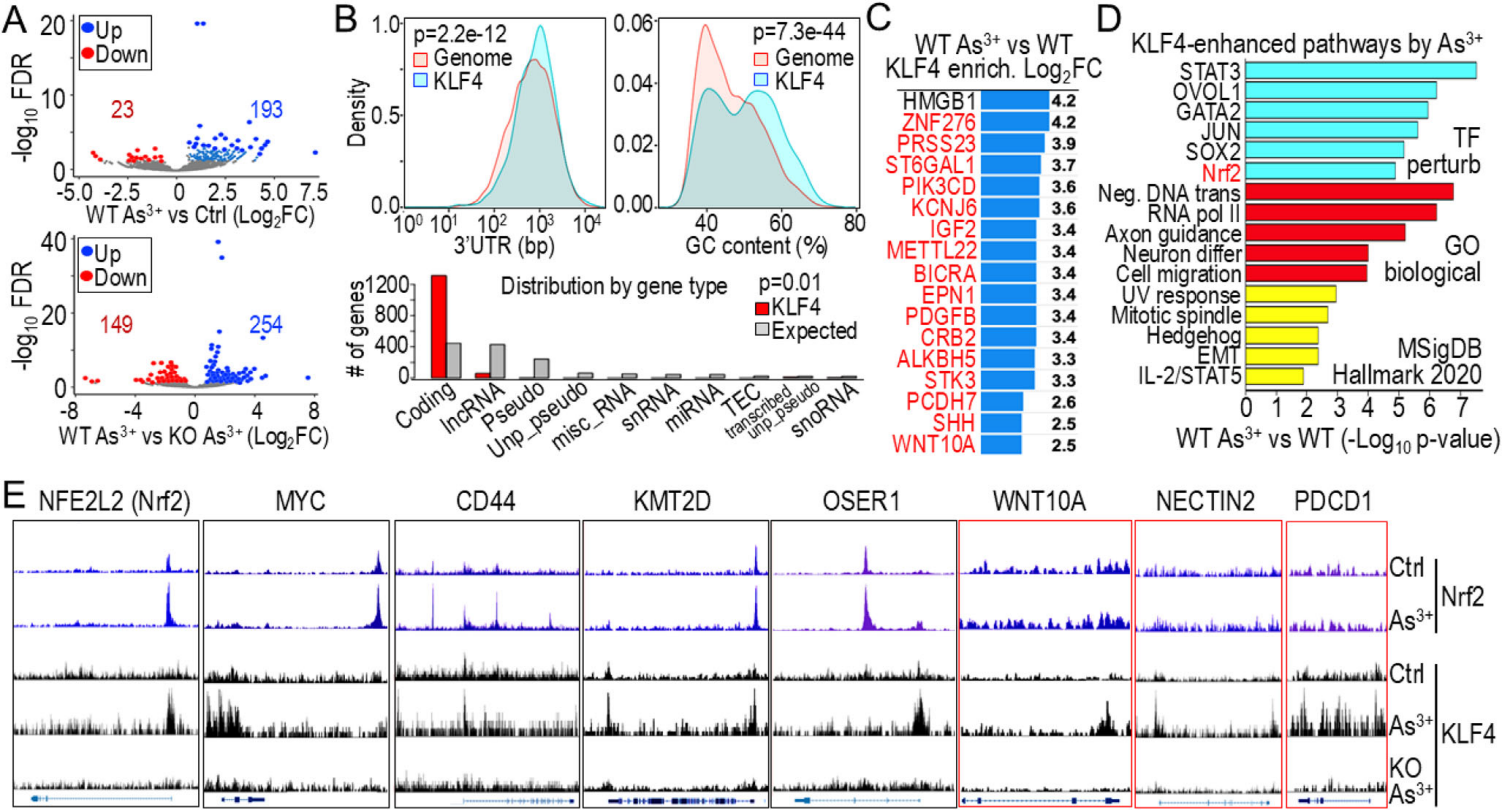
Identifying KLF4 target genes through ChIP‐seq. A) Volcano plots showing genes regulated by KLF4 in As^3+^‐treated cells versus control cells (top panel) and in As^3+^‐treated WT cells versus As^3+^‐treated Nrf2 KO cells. B) Characteristics of KLF4‐regulated genes in response to As^3+^ treatment. C) Top KLF4‐enriched genes induced by As^3+^. Genes marked in red are upregulated in As^3+^‐transformed cells. Data is shown as average log2 fold changes (n = 3 per group). D) Pathway analysis of KLF4‐regulated genes in response to As^3+^. E) KLF4 binding to Nrf2‐target genes with or without overlapping Nrf2‐binding peaks in Nrf2 ChIP‐seq data.

KLF4 is well known for its role in maintaining self‐renewal in stem cells and induced pluripotent stem cells (iPSCs). However, its transcriptional targets in differentiated cells remain unclear or controversial. ChIP‐seq analysis in WT cells revealed that As^3+^ treatment significantly increased KLF4 enrichment across thousands of genes, a process that was largely reversed in Nrf2 KO cells (**Figure** **8**A). Further analysis of genes exhibiting enhanced KLF4 enrichment in response to As^3+^ revealed a preference for genes with longer 3′‐UTR and lower GC content in their sequences (Figure 8B, top panels). Gene type distribution analysis indicated that KLF4 primarily binds to protein‐coding genes, while showing reduced affinity for long non‐coding RNAs, pseudogenes, and other non‐coding RNAs (Figure 8B, bottom panel). Interestingly, while only ~30% of KLF4‐enriched genes exhibited increased expression in As^3+^‐transformed cells, 16 out of the top 17 genes, with the exception of HMGB1, were significantly overexpressed in these cells (Figure 8C). Pathway analysis of As^3+^‐induced KLF4‐enriched genes revealed an overrepresentation of key transcription factors, including STAT3, OVOL1, GATA2, JUN, SOX2, and Nrf2, as well as functional pathways related to gene expression, cell migration, and EMT (Figure 8D). Notably, several Nrf2‐regulated genes, such as Nrf2 itself, *MYC*, *CD44*, *KMT2D*, and *OSER1* (oxidative stress responsive serine rich 1), exhibited As^3+^‐induced KLF4 binding (Figure 8E). Moreover, As^3+^ exposure led to significant KLF4 enrichment on *WNT10A* and specific immune checkpoint genes such as *NECTIN2* (CD112), and *PDCD1* (PD‐1), whose expression is Nrf2 dependent, despite the absence of detectable Nrf2 binding to these genes in ChIP‐seq analysis (Figure 8E). These findings underscore the complex regulatory landscape of KLF4 in As^3+^‐exposed cells and highlight its potential role in modulating oncogenic and immune pathways.

### 2.9 Concerted Regulation of Nrf2 and KLF4 on Enhancers

**Figure 9:**
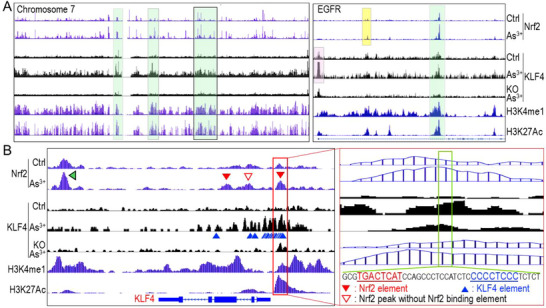
Co‐occupancy of Nrf2 and KLF4 facilitates enhancer activation. A) Genome browser tracks showing representative distribution and co‐localization of Nrf2, KLF4, H3K4me1, and H3K27Ac across chromosome 7 (left) and at the EGFR gene locus (right). Green boxes highlight regions of Nrf2 and KLF4 co‐occupy that also exhibit enhancer‐associated histone modifications (H3K4me1 and H3K27Ac), suggesting potential enhancer activity. B) Schematic representation of KLF4 self‐regulation, showing enhanced KLF4 binding within its own gene body and upstream regulatory regions. The red box highlights a region of Nrf2 and KLF4 co‐occupancy overlapping with active enhancer markers. Blue triangles denote predicted KLF4 binding elements, while red filled triangles indicate Nrf2 peaks containing ARE elements. The red unfilled triangle marks an Nrf2 peak lacking a typical ARE element. The green triangle points to the strongest Nrf2 enrichment peak, located downstream of the KLF4 gene in a region devoid of KLF4 binding or H3K27Ac signal.

At the chromosome level, we observed a tendency for As^3+^‐enhanced Nrf2 and KLF4 to co‐localize with regions exhibiting elevated levels of H3K4me1 and H3K27Ac (**Figure** **9**A). This pattern was also evident at the individual gene level, as exemplified by EGFR. Specifically, the As^3+^‐induced Nrf2 peak overlapped with the KLF4 peak within the downstream region of intron 1, where high levels of H3K4me1 and H3K27Ac were present (highlighted in green). Notably, enhancer marker levels, particularly H3K27Ac, were reduced in regions where Nrf2 (highlighted in yellow) and KLF4 (highlighted in bright pink) were not co‐localized (Figure 9A, right). This notion was further supported by the co‐localization of Nrf2, KLF4, and enhancer markers on the KLF4 gene itself. Following As^3+^ exposure, we observed increased Nrf2 binding at multiple sites, including downstream, immediately upstream, and across the gene body, spanning exon 1 to exon 3 (Figures 3B, and 9B), where over 15 KLF4‐binding elements were identified, with some forming clusters (Figure 9B, highlighted by blue triangles). Notably, the density of KLF4‐binding elements correlated proportionally with the height of KLF4 peaks in ChIP‐seq data, with the highest KLF4 clusters located near a strong Nrf2 peak containing a conserved ARE element. This alignment closely mirrored the distribution of active enhancer markers, particularly H3K27Ac (highlighted by a red box in Figure 9B). Interestingly, despite the presence of a strong As^3+^‐induced Nrf2 peak in the downstream region of the KLF4 gene (pointed by a green triangle in Figure 9B), we observed no detectable KLF4 binding peaks and only a marginal H3K27Ac peak in this region. These findings indicate that, upon Nrf2 activation, KLF4 can effectively self‐regulate its own gene expression. Furthermore, the co‐localization or spatial proximity of Nrf2 and KLF4 facilitates the establishment of active enhancers, reinforcing their concerted role in transcriptional regulation.

## 3 Discussion

We present previously undocumented evidence demonstrating that Nrf2 promotes the expression of the stemness factor KLF4 through the coordinated regulation of enhancers and KLF4 itself, further reinforcing Nrf2's pro‐carcinogenic role in cancer cells and CSCs. CRISPR‐cas9‐mediated depletion of Nrf2 resulted in a marked decrease in KLF4 expression, its nuclear translocation, and chromatin binding, as well as a reduction in As^3+^‐induced malignant transformation. ChIP‐seq analysis identified multiple Nrf2 enrichment peaks at the KLF4 gene locus, particularly under As^3+^‐induced stress conditions, with most peaks containing conserved ARE elements, validating Nrf2's direct transcriptional regulation of KLF4. Beyond direct transcriptional activation, Nrf2 enhances KLF4 expression by facilitating the establishment of enhancers at the KLF4 locus and other genomic regions, as evidenced by the partial Nrf2 dependence of KMT2D, a methyltransferase responsible for H3K4me1. Intriguingly, Nrf2 and KLF4 collaborate in the formation of active enhancers, while KLF4 further contributes to its own expression by binding to multiple KLF4 elements located within its gene body and upstream of its promoter.

As one of the original four Yamanaka factors identified for reprogramming iPSCs, KLF4 has been extensively studied in self‐renewal and lineage development of pluripotent stem cells and progenitor cells.^[^ ^36^ ^]^ However, its role as an oncogenic factor or tumor suppressor in carcinogenesis and tumor progression remains context‐dependent and controversial. One of the earliest findings supporting KLF4's oncogenic role is the attenuation of mutant Kras^G12D^‐driven pancreatic intraepithelial neoplasia (PanIN) following *Klf4* ablation in a mouse model of pancreatic cancer.^[^ ^37^ ^]^ Stress‐induced KLF4 expression has been implicated in acinar‐to‐ductal metaplasia (ADM), a precursor lesion in PanIN development. In breast cancer, KLF4 overexpression enhances metastatic migration and invasion,^[^ ^38^ ^]^ while in osteosarcoma, KLF4 is critical for chemotherapy‐induced stemness and metastasis.^[^ ^39^ ^]^ In human lung cancer, KLF4 expression varies depending on cancer type and stage. Its expression is elevated in small‐cell lung cancer (SCLC) compared to normal tissue. In non‐small cell lung cancer (NSCLC), higher KLF4 levels are observed in stages II, III, and IV compared to stage I.^[^ ^40^ ^]^ Intriguingly, some studies suggest a tumor suppressor‐like role for KLF4 in specific cancers. For example, KLF4 overexpression in murine prostate stem cells impedes malignant transformation, whereas KLF4 loss induces molecular features associated with aggressive prostate tumors.^[^ ^41^ ^]^ These paradoxical roles may be cell type‐dependent, influenced by the balance between KLF4 and other oncogenic or tumor suppressive factors. In normal cells, KLF4 may act as a tumor suppressor by inducing p21^CIP1^ (CDKN1A), but in cancerous or transformed cells, KLF4 may exert oncogenic functions by antagonizing p53 activity.^[^ ^42^ ^]^ The complexity of KLF4's function in cancer is further underscored by its structural and functional similarities to other KLF family proteins, which consists of 18 members, each responding to diverse stress signals.^[^ ^43^, ^44^ ^]^ This highlights the need for further investigations into context‐dependent KLF4 regulation and its interplay with other oncogenic and tumor‐suppressive pathways.

We and others have previously reported that Nrf2 acts as a transcriptional regulator of KLF4 in human cells.^[^ ^11^, ^45^ ^]^ However, the discovery of multiple Nrf2 binding sites, or ARE elements, within the KLF4 gene locus, exhibiting strong Nrf2 enrichment in response to As^3+^ exposure, has not been previously documented. Particularly intriguing is the presence of Nrf2‐binding clusters overlapping with the enhancer markers H3K4me1 and H3K27Ac, located 218 kb upstream of the KLF4 gene. In HeLa cells, this upstream region appears to play a crucial role in forming a topologically associating domain (TAD) that connects the distal enhancer e7 (Figure 3B) with the KLF4 promoter, as well as linking downstream proximal enhancer e1 to distal enhancer e7, thereby facilitating *KLF4* transcription (data not shown). Nrf2 knockout resulted in a marked reduction in KLF4 expression, accompanied by a decrease in enhancer markers overlapping with Nrf2 clusters, suggesting that, beyond direct transcription regulation, Nrf2 promotes KLF4 expression by activating enhancers and facilitating TAD formation. Indeed, our data indicates that Nrf2 is a key regulator of KMT2D, a methyltransferase responsible for H3K4me1 deposition and enhancer establishment.

Despite extensive research on Nrf2's role as a transcription factor, its function in enhancers and super‐enhancer regulation remains largely unexplored. Early studies showed that the Neh4 and Neh5 domains of Nrf2 physically interact with CREBBP (CBP/p300),^[^ ^46^, ^47^ ^]^ an acetyltransferase that catalyzes H3K27Ac deposition, thereby activating enhancers.^[^ ^48^ ^]^ This suggests that Nrf2 binding on ARE elements may recruit CREBBP, facilitating enhancer activation. In contrast, KLF4's association with enhancers is well‐documented, particularly in the context of iPSC reprogramming. Emerging evidence indicates that KLF4 orchestrates the topological reorganization of enhancer‐based TADs and genome‐wide enhancer rewiring,^[^ ^49^ ^]^ in addition to directly interacting with and recruiting CREBBP.^[^ ^50^ ^]^ However, in differentiated or cancer cells, KLF4 alone may be insufficient for CREBBP recruitment, enhancer activation, or chromatin architectural reorganization, suggesting the need for additional architectural factors or co‐regulators. In this context, Nrf2 emerges as a critical co‐regulator that collaborates with KLF4 in establishing and activating enhancer hubs and their associated topological structures. Our findings, demonstrating that co‐occupied Nrf2 and KLF4 regions exhibit active enhancer characteristics, strongly supports this hypothesis.

In summary, our findings demonstrate that Nrf2 not only directly promotes KLF4 expression but also enhances KLF4's chromatin‐binding, architectural functions, and role in enhancer activation and TAD reorganization. These insights provide a mechanistic explanation for the frequent association between gain‐of‐function Nrf2 mutations and aggressive tumor behavior, including drug resistance, recurrence, and metastasis. More importantly, the regulation of KLF4 by Nrf2, as discovered in our cellular models, is supported by the strong correlation between Nrf2 and KLF4 in patient survival and therapeutical response to immune checkpoint blockade in lung and other types of tumors. Thus, targeting Nrf2 emerges as a promising strategy to limit the generation and expansion of CSCs responsible for metastasis and tumor relapses. Despite these advances, our understanding of the roles of Nrf2 and KLF4 in enhancer function and chromatin architecture remains in its infancy, highlighting the urgent need for further investigation into their interactions and oncogenic roles across diverse cellular models, animal studies, and clinical settings in human cancers.

## 4 Experimental Section

### Cell Culture

The human bronchial epithelial cell line BEAS‐2B, obtained from the American Type Culture Collection (ATCC, Manassas, VA), was cultured at 37 °C in a humidified atmosphere with 5% CO_2_. The cells were grown in DMEM (Gibco, cat#11 965 092) supplemented with 5% fetal bovine serum (R&D Systems, cat#S11150), 1% penicillin‐streptomycin (Gibco, cat#15 140 122), and 1% L‐glutamine (Gibco, cat#25 030 081). The medium was refreshed every other day, and cells were passaged weekly.

The human induced pluripotent stem cell (iPSC) line Nips‐B2 (HPS0223)^[^ ^30^ ^]^ was sourced from the RIKEN Bioresource Center (Tsukuba, Japan). Cells were cultured on 60‐mm dishes pre‐coated with iMatrix‐511 (TaKaRa, cat#T304) using StemFit Basic04 Complete Medium (amsbio, cat#SF041‐001), supplemented with 80 ng/mL basic fibroblast growth factor (bFGF) and 1% penicillin/streptomycin (Gibco, cat#15 140 122). Medium was replaced daily to maintain optimal growth conditions. For subculturing, cells were enzymatically dissociated into single cells with TrypLE Select (Gibco, cat#12 563 011).

### CRISPR‐Cas9 Knockout of Nrf2

Nrf2 knockout was generated using CRISPR‐Cas9 gene editing, following the protocol described before.^[^ ^11^ ^]^ In brief, single guide RNAs (sgRNAs) targeting exon 2 of the human Nrf2 gene were designed via CRISPOR (http://crispor.tefor.net/). Two sgRNAs with high scores were chosen: sgRNA‐1 (5′‐TATTTGACTTCAGTCAGCGA‐3′) and sgRNA‐2 (5′‐GCGACGGAAAGAGTATGAGC‐3′). These double‐stranded sgRNAs were ligated into the pSpCas9‐2A‐Blast vector (Addgene, cat#118 055). BEAS‐2B cells were transfected with the constructed pSpCas9‐2A‐Nrf2 plasmid using Lipofectamine 2000 (Invitrogen, cat#11668‐018) according to the manufacturer's protocol. After blasticidin selection at 4 µg mL^−1^, ^−^20 colonies from each sgRNA transfection were analyzed by western blotting to determine Nrf2 expression. Colonies lacking detectable Nrf2 protein were classified as knockout (KO), whereas those expressing Nrf2 were designated as wild type (WT).

### *Nrf2* Overexpression

For *Nrf2* overexpression, the coding sequence (CDS) region of the human Nrf2 gene was amplified by nested PCR and subsequently cloned into the pCDH‐MSC‐coGFP‐puro lentiviral expression vector, which had been digested with XbaI (NEB, cat#R0145) and BamHI (NEB, cat#R3136). The primer sequences used for cloning are listed in Table S2 (Supporting Information). Lentiviral particles were generated by co‐transfecting HEK 293T cells with the pCDH‐*Nrf2* construct or the empty vector, along with the packaging plasmids psPAX2 and pMD2.G, using polyethylenimine (PEI, 1 mg/mL, TOCRIS, cat#7854). Viral supernatants were collected at 48 and 72 h post‐transfection and filtered through a 0.45 µm syringe filter. Nrf2 KO cells and As^3+^‐transformed Nrf2 KO cells were transduced with the viral supernatants in the presence of 8 µg/mL polybrene. After 12 h, the medium was replaced with fresh complete medium. After 72 h post‐transduction, cells were selected with 1 µg mL^−1^ puromycin for 1 week. Viable cells were then harvested for subsequent protein expression analysis.

### Development of As^3+^‐Transformed Cells

WT and Nrf2 KO BEAS‐2B cells were exposed to 0.25 µm As^3+^, while control cells received an equal volume of double‐distilled water as the vehicle. To establish As^3+^‐transformed cell models, both WT and Nrf2 KO cells underwent continuous treatment with 0.25 µm As^3+^ for 3 months. Similarly, malignant transformation of the human iPSC Nips‐B2 was induced by prolonged exposure to 0.25 µM As^3+^ for 105 consecutive days. Throughout the treatment period, culture medium was refreshed daily, and cells were passaged twice per week. Untreated Nips‐B2 cells, cultured in parallel under identical conditions, served as controls.

### Soft Agar Colony Formation Assay

A total of 5 × 10^3^ cells per well were suspended in DMEM containing 0.3% agar (BD Microbiology, cat#214 010) and plated on a base layer of 0.5% agar (prepared with 2 × DMEM) in a 6‐well plate. After 2 weeks of incubation, colonies were stained with 1.0 mg mL^−1^ MTT and viewed under a microscope.

### Subcutaneous Tumor Formation

Four‐week‐old female NOD scid gamma (NSG) mice (Jackson Laboratory, Boston, MA) were housed under federal guidelines. All procedures were approved by the Institutional Animal Care and Use Committee (IACUC) at Stony Brook University (protocol #2021‐00104). Mice were randomly assigned to four groups (n = 5 per group): WT, As^3+^‐transformed WT, Nrf2 KO, and As^3+^‐transformed Nrf2 KO. After acclimatization, each mouse received a subcutaneous injection of 2 × 10⁶ cells. Tumor growth was monitored for 8 weeks before euthanasia, after which tumor number and size were assessed. Tumor volume was calculated using the formula: *V*  =  π/6  × *L* × *W* ^2^, where *L* represents the longest diameter of the tumor mass, and *W* denotes the longest diameter perpendicular to *L*.

### Western Blotting

Cells were lysed in ice‐cold RIPA buffer with 1 mM phenylmethanesulfonyl fluoride (PMSF) (Sigma‐Aldrich, cat#P7626). Total protein extraction was performed as previously described^[^ ^11^ ^]^ and quantified using the Pierce BCA Protein Assay Kit (Thermo Scientific, cat#23 225). Equal amounts of protein (30 µg) were separated on 10% SDS‐PAGE and transferred to a polyvinylidene fluoride (PVDF) membrane (Thermo Fisher, cat#YJ382392). After blocking with 5% fat‐free milk, membranes were incubated overnight at 4 °C with primary antibodies: rabbit anti‐Nrf2 (CST, cat#12 721, 1:1000), rabbit anti‐KLF4 (CST, cat#4038, 1:1000), mouse anti‐MYC (CST, cat#2276, 1:1000), rabbit anti‐KMT2D (Abcam, cat#ab231239, 1:500), rabbit anti‐EP300 (CST, cat#86 377, 1:1000), and mouse anti‐GAPDH (CST, cat#97 166, 1:1000). Membranes were then incubated with HRP‐conjugated secondary antibodies: anti‐rabbit IgG (CST, cat#7074, 1:3000) or anti‐mouse IgG (CST, cat#7076, 1:500) for 1 h at room temperature. Protein bands were detected using the ChemiDoc MP imaging system (Bio‐Rad).

### ChIP‐qPCR

ChIP analysis of Nrf2 binding within the KLF4 gene body was performed using the Pierce Magnetic ChIP Kit (Thermo Scientific, cat#26 157). Cells were cross‐linked with 1% formaldehyde, quenched with 0.125 m glycine, lysed, and digested with MNase according to the manufacturer's protocol. The supernatant was incubated overnight at 4 °C with an Nrf2 antibody (Proteintech, cat#16396‐1‐AP, 1:200), followed by a 2 h incubation with ChIP Grade Protein A/G Magnetic Beads. After sequential washes, DNA‐protein complexes were eluted, cross‐links reversed at 65 °C for 1.5 h, and DNA extracted for qPCR analysis. IgG served as a negative control, while Anti‐RNA Polymerase II was the positive control. Primers used for ChIP‐qPCR are listed in Table S3 (Supporting Information). qPCR was carried out with SYBR Green PCR Master Mix (Thermo Fisher, cat#2 211 538) according to the manufacturer's instructions.

### Nrf2 Luciferase Reporter Assay

Nrf2 activation was assessed by the Human ARE Reporter Kit (BPS Biosciences, cat#60 514) following the manufacturer's instructions. Luciferase activities were sequentially measured using a VARIOSKAN LUX (Thermo Scientific).

### RNA Sequencing (RNA‐seq)

Total RNA was extracted from 2.0 × 10^6^ cells per sample following culture with or without 2 µm As^3+^ for 12 h, as well as from WT and Nrf2 KO BEAS‐2B cells and iPSCs chronically exposed to 0.25 µM As^3+^. RNA sequencing and library preparation were conducted using the Illumina platform by Active Motif Inc., with the human genome assembly hg19 as the reference. Transcript assembly and differential expression analysis were performed using Cufflinks, and gene expression levels were normalized based on geometric FPKMs (Fragments Per Kilobase of transcript per Million mapped reads). Differentially expressed genes (DEGs) were identified through pairwise comparisons.

### Chromatin Immunoprecipitation Sequencing (ChIP‐seq)

WT and Nrf2 KO cells (1 × 10^7^) were treated with or without 1 µm As^3+^ for 6 h before undergoing ChIP‐seq. Additionally, ChIP‐seq was performed on iPSCs that had undergone chronic exposure to 0.25 µm As^3+^. Library preparation and sequencing were conducted by Active Motif. Cells were fixed with 4% formaldehyde at 25 °C for 15 min, and crosslinking was halted with 125 mm glycine for 5 min at room temperature. After PBS washes, cells were lysed, and chromatin was sheared to an average fragment length of 300–500 bp via sonication. Genomic DNA (Input) was prepared by treating chromatin with RNase and proteinase K, followed by de‐crosslinking and ethanol precipitation. DNA concentration was measured using a NanoDrop spectrophotometer (Thermo Scientific). A 30 µg aliquot of chromatin was precleared with protein A agarose beads (Invitrogen), and ChIP was performed using 4 µg of antibodies against H3K4me1 (Thermofisher, cat#710795), H3K4me3 (Thermofisher, cat#711958), H3K9me3 (CST, cat#4658), H3K27Ac (CST, cat#8173), H3K27me3 (CST, cat#9733), H3K36me3 (CST, cat#4909), H4K20me3 (CST, cat#5737) and KLF4 (R&D Systems, cat#AF3640). After immunoprecipitation, complexes were washed, eluted with SDS buffer, and treated with RNase and proteinase K. Crosslinks were reversed by overnight incubation at 65 °C, and DNA was purified via phenol‐chloroform extraction and ethanol precipitation. Illumina sequencing libraries were prepared through end‐polishing, dA‐addition, and adapter ligation, followed by PCR amplification. The sequencing was conducted using the Illumina NextSeq 500 platform. Reads (75‐nt) were aligned to the GRCh37/hg19 or GRCh38/hg38 genome using the Burrows‐Wheeler Aligner (BWA) with default settings. Fragments were extended to 200 bp and density maps were generated in 32‐nt bins. Tag numbers were normalized via random sampling to the smallest sample size, and data were visualized using the UCSC genome browser. Raw ChIP‐seq data for Nrf2, KLF4, H3K4me1, and H3K27Ac are available in the Gene Expression Omnibus (GEO) database (accession number GSE279482, GSE145834).

### Clinical Relevance Analysis of Nrf2 in Human Cancers

The prognostic significance of Nrf2 expression was assessed using the Kaplan‐Meier Plotter (http://kmplot.com/analysis/) based on two Affymetrix probes (201146_at and 1567013_at) in lung cancer patients, with subgroup analyses by stage, smoking status, and histology. Nrf2 expression was also compared between responders and non‐responders to immune checkpoint inhibitors (anti‐PD1/CTLA‐4) across cancer types using the ROC Plotter platform (https://rocplot.com/). Genomic alterations in Nrf2 were analyzed via the NCI GDC Data Portal (https://portal.gdc.cancer.gov/), and their associations with patient survival were further investigated using TNMplot (https://tnmplot.com/analysis/).

### Pathway Enrichment Analysis

Pathway enrichment analysis for ChIP‐seq and RNA‐seq data was conducted using Enrichr (https://maayanlab.cloud/Enrichr/). For ChIP‐seq data, gene symbols or IDs derived from identified peaks were uploaded to Enrichr, where they were analyzed against gene set libraries including KEGG, Reactome, and the Human Molecular Signatures Database (MSigDB). In the case of RNA‐seq, DEGs were identified based on fold change and adjusted *p*‐values followed by enrichment analysis in Enrichr. Enrichr computed enrichment scores and *p*‐values to identify significantly enriched pathways and functional processes.

### Statistical Analysis

Quality control for the ChIP‐seq and RNA‐seq data was conducted by Active Motif (Carlsbad, CA). Statistical analyses were performed using GraphPad Prism 7 (GraphPad Software, San Diego, CA). Unless otherwise specified, all experiments were conducted independently in triplicate with at least three biological replicates. Data are presented as mean ± standard deviation (SD), unless stated otherwise. Comparisons between groups were evaluated using either Student's *t*‐test or one‐way analysis of variance (ANOVA), as appropriate. A *p*‐value of < 0.05 was considered statistically significant for all two‐sided tests.

## Conflict of Interest

The authors declare no conflict of interest.

## Author Contributions

Z. W. performed conceptualization, wrote original draft, performed methodology, software, investigation, visualization; Z.B. performed methodology, software, investigation, visualization; J.B. and Y.Q. performed methodology, investigation; W.Z., B.A., and H. J. performed methodology, data curation; J.H. and C.T. performed methodology, validation; F.C. performed conceptualization, wrote, reviewed, and edited the manuscript, performed supervision, project administration, funding acquisition.

## Supporting information



Supporting Information

## Data Availability

The data that support the findings of this study are available from the corresponding author upon reasonable request.
