# Supplementary material for: Nrf2 Drives Epigenetic Reprogramming and Acts as the Master Regulator of KLF4 Expression and Activity in Arsenic‐Induced Transformation
Source: Adv Sci (Weinh). 2025 Aug 4;12(40):e00221. doi: 10.1002/advs.202500221 (PMC12561416; doi:10.1002/advs.202500221)
Supplement: Supplementary file 1 — Supporting Information [file ADVS-12-e00221-s001.docx]

**Nrf2 drives epigenetic reprogramming and acts as the master regulator of KLF4 expression and activity in arsenic-induced transformation**

**Authors**

Ziwei Wang^1^*, Zhuoyue Bi^1^, Jessica Bamrah^1^, Yiran Qiu^1^, Wenxuan Zhang^1^, Bandar Saeed Almutairy^2^, Haoyan Ji^1^, John D. Haley^1,3^, Chitra Thakur^1^, Fei Chen^1^*

**Affiliations**

*^1^ Stony Brook Cancer Center and Department of Pathology, Renaissance School of Medicine, Stony Brook University, Lauterbur Drive, Stony Brook, NY 11794, USA*

*^2^ Department of Pharmaceutical Sciences, College of Pharmacy, Shaqra University, Shaqra 11961, Saudi Arabia*

*^3^ Stony Brook University Proteomics Center, Renaissance School of Medicine, Stony Brook University, 110 Nicolls Road, Stony Brook, NY 11794, USA*

* Correspondence should be addressed to:

Dr. Ziwei Wang, [Ziwei.Wang@stonybrook.edu](mailto:Ziwei.Wang@stonybrook.edu)

Or

Dr. Fei Chen, [Fei.Chen.1@stonybrook.edu](mailto:Fei.Chen.1@stonybrook.edu)

| **Table S1 List of 604 genes positively correlated with Nrf2 expression (Spearman’s Rho ≥ 0.5) across 1,865 human lung tumor samples**   \| **Gene name** \| **Spearman's Rho** \| **p-value** \| \| --- \| --- \| --- \| \| SEPT10 \| 0.77 \| 0.00e+00 \| \| ACAP2 \| 0.74 \| 0 \| \| RNF13 \| 0.73 \| 0 \| \| RNF6 \| 0.71 \| 3.72E-286 \| \| RCN2 \| 0.7 \| 3.05E-272 \| \| SDHD \| 0.7 \| 6.16E-279 \| \| TMEM123 \| 0.7 \| 3.68E-275 \| \| NAA50 \| 0.7 \| 3.52E-278 \| \| MMADHC \| 0.7 \| 5.84E-273 \| \| PRNP \| 0.69 \| 2.64E-262 \| \| DLD \| 0.69 \| 7.18E-262 \| \| C1D \| 0.68 \| 1.09E-251 \| \| RAP1B \| 0.68 \| 1.38E-253 \| \| SYPL1 \| 0.68 \| 1.94E-257 \| \| SWAP70 \| 0.68 \| 2.3E-249 \| \| LAMP2 \| 0.67 \| 7.64E-246 \| \| ZC3H15 \| 0.67 \| 3.25E-240 \| \| TWF1 \| 0.67 \| 4.29E-243 \| \| MFN1 \| 0.67 \| 1.56E-240 \| \| NCK1 \| 0.67 \| 1.3E-244 \| \| ATP11B \| 0.67 \| 4.35E-245 \| \| OSBPL11 \| 0.67 \| 4.66E-241 \| \| NRBF2 \| 0.67 \| 7.23E-247 \| \| PTPN12 \| 0.66 \| 2.93E-231 \| \| NPTN \| 0.66 \| 2.2E-229 \| \| XPO1 \| 0.66 \| 3.65E-234 \| \| ELF1 \| 0.66 \| 1.96E-238 \| \| CSNK1A1 \| 0.66 \| 1.52E-236 \| \| TSPYL1 \| 0.66 \| 2.6E-237 \| \| ASUN \| 0.66 \| 9.73E-238 \| \| BTG1 \| 0.65 \| 8.9E-226 \| \| DPM1 \| 0.65 \| 7.96E-226 \| \| HAT1 \| 0.65 \| 4.54E-225 \| \| NDUFB5 \| 0.65 \| 2.24E-225 \| \| FZD6 \| 0.65 \| 3.88E-221 \| \| BACH1 \| 0.65 \| 7.74E-228 \| \| PAK2 \| 0.65 \| 4.2E-220 \| \| ISCA1 \| 0.65 \| 1.39E-225 \| \| MORC3 \| 0.65 \| 3.78E-220 \| \| DCAF6 \| 0.65 \| 2.17E-226 \| \| KLF4 \| 0.65 \| 3.83E-220 \| \| RPA1 \| 0.64 \| 2.55E-218 \| \| VPS26A \| 0.64 \| 9.4E-216 \| \| PPP1R2 \| 0.64 \| 1.85E-213 \| \| ARFGAP3 \| 0.64 \| 1.05E-218 \| \| SF3B1 \| 0.64 \| 4.18E-219 \| \| TMEM131 \| 0.64 \| 7.5E-219 \| \| KDM3A \| 0.64 \| 3.48E-216 \| \| LAMTOR3 \| 0.64 \| 2.56E-218 \| \| KPNA3 \| 0.64 \| 1.13E-217 \| \| SPATA5L1 \| 0.64 \| 3.32E-211 \| \| EIF3A \| 0.63 \| 1.48E-206 \| \| CREG1 \| 0.63 \| 3.07E-209 \| \| ITGA6 \| 0.63 \| 1.05E-210 \| \| CTR9 \| 0.63 \| 1.18E-203 \| \| TDP2 \| 0.63 \| 6.3E-209 \| \| ITGAV \| 0.63 \| 1.46E-204 \| \| SSFA2 \| 0.63 \| 8.29E-208 \| \| SEC24B \| 0.63 \| 7.2E-208 \| \| EMC2 \| 0.63 \| 5.01E-209 \| \| CPOX \| 0.63 \| 1.22E-205 \| \| ZBTB11 \| 0.63 \| 3.33E-205 \| \| ADH5 \| 0.63 \| 5.2E-208 \| \| DDX18 \| 0.63 \| 4.17E-206 \| \| SPAST \| 0.63 \| 8.79E-208 \| \| TNFAIP8 \| 0.63 \| 1.58E-208 \| \| RTF1 \| 0.63 \| 1.27E-205 \| \| UBE3A \| 0.63 \| 7.64E-208 \| \| ARL8B \| 0.63 \| 8.78E-206 \| \| MFSD1 \| 0.63 \| 1.41E-210 \| \| COMMD8 \| 0.63 \| 8.36E-208 \| \| SLC35A5 \| 0.63 \| 1.58E-207 \| \| CCDC91 \| 0.63 \| 1.02E-204 \| \| EIF2AK3 \| 0.63 \| 7.82E-203 \| \| ATG3 \| 0.63 \| 1.08E-204 \| \| WDR61 \| 0.63 \| 2.66E-208 \| \| ATF1 \| 0.63 \| 4.85E-207 \| \| ANXA1 \| 0.62 \| 5.57E-197 \| \| ADD3 \| 0.62 \| 2.61E-198 \| \| EMP1 \| 0.62 \| 1.02E-200 \| \| CNIH1 \| 0.62 \| 2.22E-199 \| \| LANCL1 \| 0.62 \| 6.11E-198 \| \| ADAM9 \| 0.62 \| 1.42E-194 \| \| MARCH7 \| 0.62 \| 1.02E-200 \| \| IFNGR1 \| 0.62 \| 5.14E-202 \| \| SUCLA2 \| 0.62 \| 3.9E-200 \| \| ACVR1 \| 0.62 \| 9.9E-201 \| \| UGP2 \| 0.62 \| 4.35E-195 \| \| MAPK6 \| 0.62 \| 9.83E-195 \| \| NCKAP1 \| 0.62 \| 5.72E-200 \| \| STAM2 \| 0.62 \| 7.53E-195 \| \| CDV3 \| 0.62 \| 5.06E-199 \| \| RCOR1 \| 0.62 \| 1.25E-200 \| \| MED4 \| 0.62 \| 5.74E-200 \| \| COPS4 \| 0.62 \| 2.67E-197 \| \| NUP54 \| 0.62 \| 8.48E-199 \| \| RNF7 \| 0.62 \| 4.03E-201 \| \| GTF3C3 \| 0.62 \| 3.26E-201 \| \| FBXO3 \| 0.62 \| 4.44E-201 \| \| NMRK1 \| 0.62 \| 2.43E-198 \| \| EIF4G2 \| 0.61 \| 1.44E-193 \| \| DDX5 \| 0.61 \| 2.07E-192 \| \| HNRNPK \| 0.61 \| 6.46E-187 \| \| PPP1CC \| 0.61 \| 1.49E-189 \| \| AMD1 \| 0.61 \| 8.47E-187 \| \| ETF1 \| 0.61 \| 5.89E-189 \| \| TLK1 \| 0.61 \| 2.22E-189 \| \| SERTAD2 \| 0.61 \| 2.73E-187 \| \| USP8 \| 0.61 \| 1.57E-187 \| \| RYK \| 0.61 \| 2.02E-187 \| \| ASF1A \| 0.61 \| 3.23E-187 \| \| STRN3 \| 0.61 \| 4.02E-187 \| \| RCAN1 \| 0.61 \| 9.25E-190 \| \| HNRNPR \| 0.61 \| 1.07E-190 \| \| RNF11 \| 0.61 \| 2.57E-190 \| \| BNIP2 \| 0.61 \| 7.28E-191 \| \| TANK \| 0.61 \| 2.66E-190 \| \| EED \| 0.61 \| 1.82E-188 \| \| PRMT3 \| 0.61 \| 3.05E-188 \| \| PCNP \| 0.61 \| 1.17E-189 \| \| BTBD1 \| 0.61 \| 7.18E-188 \| \| ASNSD1 \| 0.61 \| 3.22E-193 \| \| MEX3C \| 0.61 \| 6.65E-192 \| \| PSMD10 \| 0.61 \| 2.88E-194 \| \| HNRNPM \| 0.6 \| 5.35E-183 \| \| TOMM20 \| 0.6 \| 1.81E-185 \| \| BCLAF1 \| 0.6 \| 4.9E-184 \| \| DARS \| 0.6 \| 1.66E-181 \| \| CYB5B \| 0.6 \| 8.48E-183 \| \| ETFA \| 0.6 \| 4.35E-180 \| \| CASP3 \| 0.6 \| 1.63E-182 \| \| PRPS2 \| 0.6 \| 1.94E-183 \| \| UTP18 \| 0.6 \| 8.16E-183 \| \| SYNCRIP \| 0.6 \| 2.28E-181 \| \| DR1 \| 0.6 \| 2.18E-183 \| \| TMX1 \| 0.6 \| 1.28E-181 \| \| MTDH \| 0.6 \| 3.96E-180 \| \| WNT5A \| 0.6 \| 1.82E-185 \| \| ENOPH1 \| 0.6 \| 6.51E-186 \| \| OSTM1 \| 0.6 \| 7.83E-186 \| \| RINT1 \| 0.6 \| 6.16E-184 \| \| IMPACT \| 0.6 \| 1.2E-183 \| \| MED17 \| 0.6 \| 4.99E-182 \| \| LIN7C \| 0.6 \| 9.13E-185 \| \| C6orf120 \| 0.6 \| 5.9E-181 \| \| EOGT \| 0.6 \| 7.93E-182 \| \| CSGALNACT2 \| 0.6 \| 3.02E-180 \| \| PTGES3 \| 0.59 \| 2.7E-175 \| \| PGRMC1 \| 0.59 \| 1.51E-177 \| \| MBNL1 \| 0.59 \| 1.06E-175 \| \| ATP6AP2 \| 0.59 \| 6.47E-175 \| \| ZFR \| 0.59 \| 6.47E-178 \| \| AASDHPPT \| 0.59 \| 3.76E-174 \| \| ADAM10 \| 0.59 \| 2.45E-173 \| \| VAMP7 \| 0.59 \| 5.69E-175 \| \| NSMAF \| 0.59 \| 2.01E-172 \| \| DCK \| 0.59 \| 9.34E-177 \| \| IFI16 \| 0.59 \| 1.5E-175 \| \| ASAP2 \| 0.59 \| 5.99E-174 \| \| SERINC1 \| 0.59 \| 2.52E-178 \| \| ELOVL5 \| 0.59 \| 1.9E-177 \| \| CLDND1 \| 0.59 \| 2.34E-175 \| \| STAG2 \| 0.59 \| 1.05E-173 \| \| PDCD10 \| 0.59 \| 2.79E-178 \| \| ABHD3 \| 0.59 \| 1.52E-175 \| \| ATP10D \| 0.59 \| 4.46E-177 \| \| TMEM165 \| 0.59 \| 7.4E-176 \| \| C6orf211 \| 0.59 \| 4.76E-172 \| \| PELI1 \| 0.59 \| 3.3E-172 \| \| FAM208B \| 0.59 \| 1.02E-175 \| \| PLEKHF2 \| 0.59 \| 4.7E-177 \| \| MTERFD1 \| 0.59 \| 1.33E-176 \| \| FAIM \| 0.59 \| 8.42E-173 \| \| PRPF18 \| 0.59 \| 9.63E-177 \| \| ADSS \| 0.59 \| 7.55E-174 \| \| FBXO11 \| 0.59 \| 1.81E-177 \| \| RRN3 \| 0.59 \| 5.62E-177 \| \| SMNDC1 \| 0.58 \| 6.35E-167 \| \| FNTA \| 0.58 \| 1.56E-168 \| \| SET \| 0.58 \| 2.48E-170 \| \| RAB11A \| 0.58 \| 1.98E-169 \| \| HNRNPA0 \| 0.58 \| 5.92E-171 \| \| PPP2CB \| 0.58 \| 1.29E-165 \| \| GJA1 \| 0.58 \| 2.35E-167 \| \| JOSD1 \| 0.58 \| 1.74E-170 \| \| UBE2A \| 0.58 \| 4.94E-165 \| \| SPTLC1 \| 0.58 \| 5.77E-171 \| \| PLSCR1 \| 0.58 \| 9.12E-171 \| \| ACTL6A \| 0.58 \| 7.61E-165 \| \| BCAS2 \| 0.58 \| 1.73E-165 \| \| AGL \| 0.58 \| 2.39E-165 \| \| HPS5 \| 0.58 \| 3.08E-165 \| \| SLC35A3 \| 0.58 \| 3.99E-168 \| \| ATP1B3 \| 0.58 \| 4.4E-169 \| \| YIPF4 \| 0.58 \| 1.3E-171 \| \| MBD4 \| 0.58 \| 1.76E-165 \| \| CASP1 \| 0.58 \| 9.98E-167 \| \| DYNC1I2 \| 0.58 \| 4.4E-165 \| \| MCFD2 \| 0.58 \| 1.52E-170 \| \| SPG20 \| 0.58 \| 6.41E-166 \| \| METAP1 \| 0.58 \| 3.64E-167 \| \| RND3 \| 0.58 \| 1.27E-165 \| \| CAMSAP2 \| 0.58 \| 5.65E-169 \| \| TBC1D9 \| 0.58 \| 4.09E-165 \| \| SGMS1 \| 0.58 \| 2.9E-170 \| \| PIKFYVE \| 0.58 \| 1.35E-171 \| \| DPY19L4 \| 0.58 \| 4.92E-167 \| \| GOLGA7 \| 0.58 \| 6.83E-169 \| \| AMZ2 \| 0.58 \| 7.99E-165 \| \| OXR1 \| 0.58 \| 6.49E-168 \| \| REV1 \| 0.58 \| 4.62E-171 \| \| RNF138 \| 0.58 \| 1.55E-166 \| \| C12orf5 \| 0.58 \| 8.74E-171 \| \| CASP8AP2 \| 0.58 \| 1.26E-171 \| \| TRA2B \| 0.57 \| 9.76E-162 \| \| UBA2 \| 0.57 \| 5.38E-158 \| \| PSMA2 \| 0.57 \| 8.15E-158 \| \| PPP1R12A \| 0.57 \| 1.11E-162 \| \| SMC4 \| 0.57 \| 6.07E-158 \| \| EIF1B \| 0.57 \| 4.53E-161 \| \| SGK1 \| 0.57 \| 4.18E-160 \| \| CLIP1 \| 0.57 \| 6.68E-158 \| \| RB1CC1 \| 0.57 \| 6.68E-158 \| \| PSMD12 \| 0.57 \| 5.08E-164 \| \| RAP1A \| 0.57 \| 5.92E-160 \| \| SRPK2 \| 0.57 \| 1.96E-161 \| \| CAPN7 \| 0.57 \| 5.52E-163 \| \| UNC50 \| 0.57 \| 1.51E-159 \| \| RAB21 \| 0.57 \| 1.57E-159 \| \| ME1 \| 0.57 \| 1.67E-163 \| \| RPS6KB1 \| 0.57 \| 2.59E-163 \| \| SRSF10 \| 0.57 \| 2.27E-161 \| \| ST13 \| 0.57 \| 9.62E-161 \| \| SRSF3 \| 0.57 \| 3.04E-162 \| \| G3BP2 \| 0.57 \| 8.39E-164 \| \| ABI1 \| 0.57 \| 8.4E-161 \| \| FBXL4 \| 0.57 \| 3.64E-160 \| \| CD44 \| 0.57 \| 1.58E-159 \| \| SUN1 \| 0.57 \| 1.39E-158 \| \| CRKL \| 0.57 \| 2.49E-163 \| \| RBL2 \| 0.57 \| 9.66E-164 \| \| LYPLA1 \| 0.57 \| 2.61E-159 \| \| PPM1B \| 0.57 \| 3.8E-158 \| \| PAQR3 \| 0.57 \| 1.15E-163 \| \| DENND4A \| 0.57 \| 4.34E-163 \| \| SERBP1 \| 0.57 \| 1.09E-163 \| \| SLC38A2 \| 0.57 \| 3.24E-159 \| \| SCPEP1 \| 0.57 \| 1.89E-159 \| \| KRCC1 \| 0.57 \| 6.83E-162 \| \| RBM7 \| 0.57 \| 3.17E-164 \| \| ZNF277 \| 0.57 \| 6.85E-159 \| \| NOC3L \| 0.57 \| 1.6E-164 \| \| NIP7 \| 0.57 \| 1.66E-162 \| \| KIF16B \| 0.57 \| 1.45E-163 \| \| C11orf58 \| 0.56 \| 2.23E-157 \| \| ACTR2 \| 0.56 \| 4.63E-157 \| \| PLS3 \| 0.56 \| 4.2E-153 \| \| NDUFA5 \| 0.56 \| 9.06E-158 \| \| GOLGA4 \| 0.56 \| 2.22E-153 \| \| PSMC6 \| 0.56 \| 2.9E-152 \| \| CCNC \| 0.56 \| 3.28E-152 \| \| RIOK3 \| 0.56 \| 2.4E-157 \| \| RSRC2 \| 0.56 \| 3.9E-152 \| \| DUSP11 \| 0.56 \| 5.25E-155 \| \| HPRT1 \| 0.56 \| 3.1E-156 \| \| WTAP \| 0.56 \| 3.59E-155 \| \| STXBP3 \| 0.56 \| 4.78E-152 \| \| SPG11 \| 0.56 \| 4.81E-157 \| \| NFIL3 \| 0.56 \| 8.51E-154 \| \| PTGER4 \| 0.56 \| 1.02E-154 \| \| MAP3K8 \| 0.56 \| 4.18E-152 \| \| CNBP \| 0.56 \| 2.01E-155 \| \| SMARCA2 \| 0.56 \| 6.51E-153 \| \| TBPL1 \| 0.56 \| 4.92E-152 \| \| CYCS \| 0.56 \| 4.65E-155 \| \| SEC62 \| 0.56 \| 2.53E-155 \| \| UBA3 \| 0.56 \| 4.56E-156 \| \| USP15 \| 0.56 \| 2.77E-155 \| \| RDX \| 0.56 \| 2.94E-154 \| \| AZIN1 \| 0.56 \| 1.46E-155 \| \| DNAJC13 \| 0.56 \| 5.1E-156 \| \| SMCHD1 \| 0.56 \| 2.45E-154 \| \| REST \| 0.56 \| 6.99E-155 \| \| ATP2B1 \| 0.56 \| 5.81E-152 \| \| PIK3C2A \| 0.56 \| 1.93E-153 \| \| AP5M1 \| 0.56 \| 1.05E-157 \| \| TRAPPC11 \| 0.56 \| 9.14E-153 \| \| USP16 \| 0.56 \| 1.31E-156 \| \| TFB2M \| 0.56 \| 1.72E-152 \| \| KCTD9 \| 0.56 \| 1.39E-155 \| \| C5orf28 \| 0.56 \| 4.16E-154 \| \| CDK17 \| 0.56 \| 9.63E-152 \| \| HNRNPC \| 0.55 \| 6.14E-147 \| \| EIF4A2 \| 0.55 \| 2.35E-145 \| \| DEK \| 0.55 \| 4.52E-148 \| \| PCNA \| 0.55 \| 3.31E-145 \| \| NCBP2 \| 0.55 \| 2.37E-147 \| \| CLIC4 \| 0.55 \| 1.43E-148 \| \| COPS5 \| 0.55 \| 4.79E-147 \| \| ATMIN \| 0.55 \| 2.01E-148 \| \| NAE1 \| 0.55 \| 2.87E-145 \| \| USP1 \| 0.55 \| 8.27E-145 \| \| PPIP5K2 \| 0.55 \| 5.04E-145 \| \| PPP2R5E \| 0.55 \| 1.73E-147 \| \| CUL5 \| 0.55 \| 1.93E-148 \| \| KLHL20 \| 0.55 \| 2.58E-150 \| \| DBF4 \| 0.55 \| 3.02E-149 \| \| PIK3C3 \| 0.55 \| 1.15E-148 \| \| PIK3CA \| 0.55 \| 1.18E-149 \| \| PPM1D \| 0.55 \| 4.01E-145 \| \| CD58 \| 0.55 \| 3.29E-147 \| \| HSPH1 \| 0.55 \| 1.16E-148 \| \| UBE2V2 \| 0.55 \| 3.98E-146 \| \| SMC3 \| 0.55 \| 2.96E-150 \| \| MED21 \| 0.55 \| 3.01E-148 \| \| ORC3 \| 0.55 \| 1.27E-148 \| \| ZFAND5 \| 0.55 \| 1.44E-150 \| \| CALD1 \| 0.55 \| 2.07E-145 \| \| NEK7 \| 0.55 \| 1.94E-146 \| \| OSBPL8 \| 0.55 \| 1.32E-145 \| \| RRAS2 \| 0.55 \| 2.68E-150 \| \| PIK3R4 \| 0.55 \| 1.09E-150 \| \| RBBP6 \| 0.55 \| 5.55E-146 \| \| SERINC5 \| 0.55 \| 2.6E-149 \| \| SEC23A \| 0.55 \| 7.7E-146 \| \| SLC38A6 \| 0.55 \| 9.06E-146 \| \| UBQLN2 \| 0.55 \| 3.22E-150 \| \| MORF4L1 \| 0.55 \| 1.85E-149 \| \| HECA \| 0.55 \| 2.99E-145 \| \| MCM9 \| 0.55 \| 3.17E-149 \| \| DPP8 \| 0.55 \| 2.14E-148 \| \| BNIP3L \| 0.55 \| 6.43E-147 \| \| CDC14B \| 0.55 \| 3.75E-145 \| \| MIS12 \| 0.55 \| 1.06E-145 \| \| JMJD1C \| 0.55 \| 1.4E-146 \| \| MTM1 \| 0.55 \| 4.09E-148 \| \| LAPTM4A \| 0.54 \| 5.02E-139 \| \| TMEM66 \| 0.54 \| 1.06E-138 \| \| STRAP \| 0.54 \| 1.14E-139 \| \| MDH1 \| 0.54 \| 3.98E-144 \| \| DDX3X \| 0.54 \| 8.54E-139 \| \| SRP9 \| 0.54 \| 3.02E-141 \| \| SH3BGRL \| 0.54 \| 1.2E-139 \| \| MYL12A \| 0.54 \| 5.55E-139 \| \| UBE3C \| 0.54 \| 2.69E-144 \| \| BIRC2 \| 0.54 \| 3.21E-139 \| \| HMGCR \| 0.54 \| 2.02E-143 \| \| CDC23 \| 0.54 \| 3.53E-139 \| \| MSH6 \| 0.54 \| 1.69E-139 \| \| PDHX \| 0.54 \| 1.39E-140 \| \| CAV2 \| 0.54 \| 7.79E-144 \| \| PSMA4 \| 0.54 \| 2.21E-141 \| \| GALNT3 \| 0.54 \| 1.85E-144 \| \| PRPSAP2 \| 0.54 \| 3.33E-140 \| \| KLF9 \| 0.54 \| 2.1E-140 \| \| TUBGCP3 \| 0.54 \| 1.06E-139 \| \| USP46 \| 0.54 \| 4.04E-141 \| \| RNGTT \| 0.54 \| 1.48E-142 \| \| RP2 \| 0.54 \| 2.72E-143 \| \| TAF1A \| 0.54 \| 6.32E-141 \| \| SCAF11 \| 0.54 \| 1.03E-139 \| \| REV3L \| 0.54 \| 2.89E-141 \| \| SRP72 \| 0.54 \| 4.45E-143 \| \| PEX2 \| 0.54 \| 3.99E-143 \| \| MYO1B \| 0.54 \| 5.05E-140 \| \| DESI2 \| 0.54 \| 1.26E-141 \| \| ANKMY2 \| 0.54 \| 7.37E-140 \| \| DCUN1D4 \| 0.54 \| 4.43E-139 \| \| ATF2 \| 0.54 \| 6.44E-139 \| \| FRMD4B \| 0.54 \| 7.23E-143 \| \| BICD2 \| 0.54 \| 4.47E-144 \| \| TMEM251 \| 0.54 \| 1.64E-144 \| \| TNFSF10 \| 0.54 \| 3.01E-142 \| \| JAG1 \| 0.54 \| 2.69E-142 \| \| RSL24D1 \| 0.54 \| 1.31E-140 \| \| ERBB2IP \| 0.54 \| 1.36E-142 \| \| ERGIC2 \| 0.54 \| 3.12E-141 \| \| PRKD3 \| 0.54 \| 2.37E-139 \| \| MAP4K3 \| 0.54 \| 3.11E-143 \| \| RCBTB1 \| 0.54 \| 5.41E-139 \| \| ACTR6 \| 0.54 \| 1.51E-142 \| \| VPS54 \| 0.54 \| 4.8E-144 \| \| QTRTD1 \| 0.54 \| 3.66E-139 \| \| RNF219 \| 0.54 \| 1.18E-138 \| \| METTL5 \| 0.54 \| 2.29E-143 \| \| ZNF146 \| 0.53 \| 1.11E-136 \| \| RAD21 \| 0.53 \| 1.73E-138 \| \| BZW1 \| 0.53 \| 5.87E-136 \| \| PAFAH1B1 \| 0.53 \| 1.6E-135 \| \| PALLD \| 0.53 \| 7.52E-136 \| \| HADHB \| 0.53 \| 8.61E-137 \| \| TAF7 \| 0.53 \| 2.27E-137 \| \| IDH1 \| 0.53 \| 2.84E-133 \| \| LUM \| 0.53 \| 2.9E-136 \| \| GBAS \| 0.53 \| 1.55E-138 \| \| FAM3C \| 0.53 \| 2.08E-136 \| \| ACSL1 \| 0.53 \| 9.42E-133 \| \| IFRD1 \| 0.53 \| 1.08E-135 \| \| DLG1 \| 0.53 \| 8.69E-137 \| \| MRPL19 \| 0.53 \| 7.8E-136 \| \| BPGM \| 0.53 \| 3.45E-136 \| \| STAM \| 0.53 \| 9.25E-138 \| \| FDX1 \| 0.53 \| 2.04E-134 \| \| IL1RAP \| 0.53 \| 7.18E-133 \| \| SLBP \| 0.53 \| 1.08E-132 \| \| EIF3J \| 0.53 \| 1.06E-135 \| \| HNRNPH3 \| 0.53 \| 2.9E-137 \| \| ANAPC13 \| 0.53 \| 6.49E-137 \| \| CDKN1B \| 0.53 \| 1.15E-137 \| \| SNAP23 \| 0.53 \| 7.04E-137 \| \| MSMO1 \| 0.53 \| 7.32E-135 \| \| DNAJA2 \| 0.53 \| 9.31E-136 \| \| WAPAL \| 0.53 \| 4.52E-133 \| \| GNG12 \| 0.53 \| 4.06E-137 \| \| PICALM \| 0.53 \| 5.66E-135 \| \| UBE2E1 \| 0.53 \| 2.97E-137 \| \| DOCK9 \| 0.53 \| 7.79E-138 \| \| DHX29 \| 0.53 \| 3.21E-137 \| \| FUBP3 \| 0.53 \| 1.66E-137 \| \| KRT10 \| 0.53 \| 1.48E-137 \| \| F2RL1 \| 0.53 \| 3.02E-138 \| \| MTMR6 \| 0.53 \| 3.96E-134 \| \| TMBIM1 \| 0.53 \| 3.4E-135 \| \| LIMA1 \| 0.53 \| 5.75E-133 \| \| KCTD3 \| 0.53 \| 1.24E-137 \| \| MAT2B \| 0.53 \| 3.36E-133 \| \| GPN3 \| 0.53 \| 9.1E-135 \| \| TMEM14A \| 0.53 \| 3.55E-136 \| \| RPAP3 \| 0.53 \| 5.55E-138 \| \| MED23 \| 0.53 \| 8.92E-136 \| \| DSE \| 0.53 \| 5.11E-134 \| \| C2orf47 \| 0.53 \| 5.41E-133 \| \| CEP63 \| 0.53 \| 1.21E-132 \| \| SLC25A32 \| 0.53 \| 2.55E-133 \| \| SUB1 \| 0.53 \| 3.98E-133 \| \| EXOC1 \| 0.53 \| 1.06E-136 \| \| RAN \| 0.52 \| 4.84E-132 \| \| CD9 \| 0.52 \| 3.96E-131 \| \| GNAI3 \| 0.52 \| 1.9E-129 \| \| CAPZA2 \| 0.52 \| 6.08E-130 \| \| DDX1 \| 0.52 \| 1.71E-127 \| \| DHX15 \| 0.52 \| 1.5E-129 \| \| PSMD7 \| 0.52 \| 3.74E-127 \| \| CPNE3 \| 0.52 \| 1.75E-130 \| \| WWTR1 \| 0.52 \| 9.7E-130 \| \| CUL4B \| 0.52 \| 1.11E-128 \| \| EIF3M \| 0.52 \| 1.91E-127 \| \| SNRPD3 \| 0.52 \| 4.25E-131 \| \| TOB1 \| 0.52 \| 7.53E-129 \| \| DNTTIP2 \| 0.52 \| 1.57E-130 \| \| RB1 \| 0.52 \| 3.57E-131 \| \| AKAP11 \| 0.52 \| 1.08E-128 \| \| FGFR2 \| 0.52 \| 9.41E-128 \| \| COBLL1 \| 0.52 \| 1.86E-129 \| \| COIL \| 0.52 \| 1.68E-132 \| \| FZD7 \| 0.52 \| 1.3E-131 \| \| DNAJB4 \| 0.52 \| 2.7E-128 \| \| AK6 \| 0.52 \| 1.64E-132 \| \| HIVEP1 \| 0.52 \| 9.09E-128 \| \| ENOX2 \| 0.52 \| 4.9E-128 \| \| CLPX \| 0.52 \| 3.04E-127 \| \| POLE3 \| 0.52 \| 2.93E-130 \| \| CYFIP1 \| 0.52 \| 1.74E-128 \| \| KLF5 \| 0.52 \| 6.85E-128 \| \| GHITM \| 0.52 \| 2.88E-131 \| \| NAB1 \| 0.52 \| 2.86E-131 \| \| PRKRIR \| 0.52 \| 4.7E-127 \| \| CETN3 \| 0.52 \| 4.89E-129 \| \| CHUK \| 0.52 \| 4.93E-132 \| \| NCOA4 \| 0.52 \| 1.57E-127 \| \| ATP5F1 \| 0.52 \| 1.69E-132 \| \| UBE2D1 \| 0.52 \| 3.12E-128 \| \| ARHGAP19 \| 0.52 \| 7.34E-130 \| \| PGAP1 \| 0.52 \| 5.64E-130 \| \| RSBN1 \| 0.52 \| 3.61E-128 \| \| TCEA1 \| 0.52 \| 7.76E-128 \| \| ITM2B \| 0.52 \| 5.1E-130 \| \| PPA1 \| 0.52 \| 5.68E-129 \| \| LAP3 \| 0.52 \| 2.37E-130 \| \| PHF3 \| 0.52 \| 5.37E-129 \| \| WBP5 \| 0.52 \| 4.63E-128 \| \| WDR11 \| 0.52 \| 4.1E-129 \| \| NFYB \| 0.52 \| 5.19E-130 \| \| RPF1 \| 0.52 \| 7.6E-131 \| \| TBK1 \| 0.52 \| 4.06E-132 \| \| LPAR6 \| 0.52 \| 1.18E-130 \| \| NUP37 \| 0.52 \| 2.66E-131 \| \| RAB7L1 \| 0.52 \| 7.8E-130 \| \| TRMT11 \| 0.52 \| 2.94E-127 \| \| TEFM \| 0.52 \| 8.11E-131 \| \| SOWAHC \| 0.52 \| 1.16E-128 \| \| MYL12B \| 0.52 \| 2.79E-131 \| \| PRKAR1A \| 0.51 \| 1.59E-123 \| \| VBP1 \| 0.51 \| 9.54E-124 \| \| OAT \| 0.51 \| 1.17E-125 \| \| INSIG1 \| 0.51 \| 4.66E-126 \| \| GALNT1 \| 0.51 \| 2.41E-125 \| \| RYBP \| 0.51 \| 8.58E-122 \| \| PLK2 \| 0.51 \| 8.66E-127 \| \| ERLIN1 \| 0.51 \| 1.06E-122 \| \| EPS8 \| 0.51 \| 8.72E-125 \| \| PSMD6 \| 0.51 \| 1.45E-123 \| \| NNT \| 0.51 \| 2.8E-126 \| \| CEBPZ \| 0.51 \| 1.9E-121 \| \| CDC40 \| 0.51 \| 3.59E-123 \| \| MFAP1 \| 0.51 \| 3.66E-126 \| \| ZNF217 \| 0.51 \| 1.17E-125 \| \| KIN \| 0.51 \| 5.57E-123 \| \| MRPL3 \| 0.51 \| 4.09E-122 \| \| SEC23IP \| 0.51 \| 1.43E-121 \| \| CRY1 \| 0.51 \| 1.32E-125 \| \| UBXN4 \| 0.51 \| 1.29E-123 \| \| XPOT \| 0.51 \| 5.68E-126 \| \| MICU2 \| 0.51 \| 1.38E-124 \| \| PTPN11 \| 0.51 \| 1.48E-126 \| \| UFL1 \| 0.51 \| 7.12E-122 \| \| SNX4 \| 0.51 \| 2E-126 \| \| NUP160 \| 0.51 \| 1.12E-123 \| \| LARP4 \| 0.51 \| 4.97E-122 \| \| KIAA1033 \| 0.51 \| 5.58E-124 \| \| STX6 \| 0.51 \| 2.56E-123 \| \| DCP2 \| 0.51 \| 1.35E-126 \| \| ACKR3 \| 0.51 \| 2.9E-123 \| \| MTMR1 \| 0.51 \| 8.41E-122 \| \| C1QBP \| 0.51 \| 3.56E-126 \| \| GGCT \| 0.51 \| 6.39E-123 \| \| RGS1 \| 0.51 \| 8.35E-123 \| \| FASTKD2 \| 0.51 \| 2.65E-123 \| \| CMPK1 \| 0.51 \| 6.32E-124 \| \| C16orf80 \| 0.51 \| 2.44E-123 \| \| SMAP1 \| 0.51 \| 2.45E-126 \| \| ISOC1 \| 0.51 \| 1.32E-122 \| \| ZBED5 \| 0.51 \| 1.07E-122 \| \| HEBP1 \| 0.51 \| 1.29E-123 \| \| PSMG2 \| 0.51 \| 1.18E-124 \| \| DCUN1D1 \| 0.51 \| 3.66E-124 \| \| LEMD3 \| 0.51 \| 3.88E-123 \| \| PSD3 \| 0.51 \| 6.16E-126 \| \| KIAA1551 \| 0.51 \| 5.91E-125 \| \| NFU1 \| 0.51 \| 1.21E-123 \| \| SLC30A5 \| 0.51 \| 3.86E-122 \| \| MFF \| 0.51 \| 1.31E-123 \| \| FASTKD3 \| 0.51 \| 1.31E-125 \| \| DDX47 \| 0.51 \| 1.02E-126 \| \| TM2D3 \| 0.51 \| 2.48E-125 \| \| ADAMTS1 \| 0.51 \| 5.36E-125 \| \| TMEM135 \| 0.51 \| 4.07E-126 \| \| CBX3 \| 0.5 \| 7.56E-118 \| \| UBE2D3 \| 0.5 \| 9.67E-117 \| \| ARHGEF12 \| 0.5 \| 4.55E-118 \| \| CLINT1 \| 0.5 \| 5.36E-117 \| \| ADNP \| 0.5 \| 1.87E-120 \| \| PPP2R5C \| 0.5 \| 1.93E-117 \| \| CCT2 \| 0.5 \| 7.97E-121 \| \| RALB \| 0.5 \| 1.32E-118 \| \| TMED5 \| 0.5 \| 3.27E-116 \| \| DNAJB9 \| 0.5 \| 3.58E-118 \| \| NOL7 \| 0.5 \| 6.02E-121 \| \| BCL6 \| 0.5 \| 2E-116 \| \| SCAF8 \| 0.5 \| 1.63E-119 \| \| CD2AP \| 0.5 \| 1.83E-117 \| \| C5orf22 \| 0.5 \| 8.65E-118 \| \| WDR47 \| 0.5 \| 4.4E-117 \| \| E2F6 \| 0.5 \| 7.38E-117 \| \| ATXN7 \| 0.5 \| 6.82E-120 \| \| NEK4 \| 0.5 \| 2.98E-121 \| \| DPYD \| 0.5 \| 5.03E-119 \| \| PIGF \| 0.5 \| 3.75E-118 \| \| ANK3 \| 0.5 \| 1.26E-119 \| \| GTF2B \| 0.5 \| 7.16E-121 \| \| OSBPL1A \| 0.5 \| 1.89E-116 \| \| PDIA6 \| 0.5 \| 3.62E-121 \| \| ATP6V1G1 \| 0.5 \| 2.99E-119 \| \| CLNS1A \| 0.5 \| 2.23E-119 \| \| ME2 \| 0.5 \| 4.03E-116 \| \| GNAI1 \| 0.5 \| 7.3E-120 \| \| RIN2 \| 0.5 \| 2.61E-120 \| \| HMGN4 \| 0.5 \| 2.86E-120 \| \| ATR \| 0.5 \| 5.99E-116 \| \| AKAP9 \| 0.5 \| 3.37E-117 \| \| RTN4 \| 0.5 \| 8E-119 \| \| KIDINS220 \| 0.5 \| 1.22E-118 \| \| PSMD14 \| 0.5 \| 1.61E-117 \| \| CERS6 \| 0.5 \| 6.92E-119 \| \| USP33 \| 0.5 \| 7.68E-117 \| \| WEE1 \| 0.5 \| 5.3E-117 \| \| TUG1 \| 0.5 \| 5.12E-116 \| \| C2CD5 \| 0.5 \| 3.67E-117 \| \| C12orf29 \| 0.5 \| 2.73E-116 \| \| ATP5A1 \| 0.5 \| 3.82E-117 \| \| WBP11 \| 0.5 \| 2.86E-120 \| \| CDC27 \| 0.5 \| 4.18E-120 \| \| IARS2 \| 0.5 \| 8.36E-120 \| \| ZC3H7A \| 0.5 \| 5.92E-117 \| \| TRAPPC13 \| 0.5 \| 2.59E-118 \| \| RNF111 \| 0.5 \| 7.14E-118 \| \| C1GALT1C1 \| 0.5 \| 5.31E-120 \| \| C11orf73 \| 0.5 \| 2.04E-117 \| \| SAMSN1 \| 0.5 \| 3.21E-116 \| \| PPA2 \| 0.5 \| 4.5E-121 \| \| TRMT61B \| 0.5 \| 1.52E-119 \| \| RAB9A \| 0.5 \| 3.01E-121 \| \| SEH1L \| 0.5 \| 1.38E-119 \| \| NOL11 \| 0.5 \| 6.26E-118 \|   **Table S2 Primers for amplifying the CDS region of *Nrf2***   \|  \| **Sequence (5’-3’)** \| \| --- \| --- \| \| Nrf2-nest-PCR F \| TGCTTTATAGCGTGCAAACC \| \| Nrf2-nest-PCR R \| TAGTTTTGGCTATGATTTTGCA \| \| Nrf2-XbaI-PCR F \| GCCTCTAGAATGGATTTGATTGACATACTTTGGA \| \| Nrf2-BmaHI-PCR R \| GCCGGATCCCTAGTTTTTCTTAACATCTGG \|   **Table S3 Gene-specific primers for Nrf2 binding elements on the *KLF* locus** | | |
| --- | --- | --- | --- | --- | --- | --- | --- | --- | --- | --- | --- | --- | --- | --- | --- | --- | --- | --- | --- | --- | --- | --- | --- | --- | --- | --- | --- | --- | --- | --- | --- | --- | --- | --- | --- | --- | --- | --- | --- | --- | --- | --- | --- | --- | --- | --- | --- | --- | --- | --- | --- | --- | --- | --- | --- | --- | --- | --- | --- | --- | --- | --- | --- | --- | --- | --- | --- | --- | --- | --- | --- | --- | --- | --- | --- | --- | --- | --- | --- | --- | --- | --- | --- | --- | --- | --- | --- | --- | --- | --- | --- | --- | --- | --- | --- | --- | --- | --- | --- | --- | --- | --- | --- | --- | --- | --- | --- | --- | --- | --- | --- | --- | --- | --- | --- | --- | --- | --- | --- | --- | --- | --- | --- | --- | --- | --- | --- | --- | --- | --- | --- | --- | --- | --- | --- | --- | --- | --- | --- | --- | --- | --- | --- | --- | --- | --- | --- | --- | --- | --- | --- | --- | --- | --- | --- | --- | --- | --- | --- | --- | --- | --- | --- | --- | --- | --- | --- | --- | --- | --- | --- | --- | --- | --- | --- | --- | --- | --- | --- | --- | --- | --- | --- | --- | --- | --- | --- | --- | --- | --- | --- | --- | --- | --- | --- | --- | --- | --- | --- | --- | --- | --- | --- | --- | --- | --- | --- | --- | --- | --- | --- | --- | --- | --- | --- | --- | --- | --- | --- | --- | --- | --- | --- | --- | --- | --- | --- | --- | --- | --- | --- | --- | --- | --- | --- | --- | --- | --- | --- | --- | --- | --- | --- | --- | --- | --- | --- | --- | --- | --- | --- | --- | --- | --- | --- | --- | --- | --- | --- | --- | --- | --- | --- | --- | --- | --- | --- | --- | --- | --- | --- | --- | --- | --- | --- | --- | --- | --- | --- | --- | --- | --- | --- | --- | --- | --- | --- | --- | --- | --- | --- | --- | --- | --- | --- | --- | --- | --- | --- | --- | --- | --- | --- | --- | --- | --- | --- | --- | --- | --- | --- | --- | --- | --- | --- | --- | --- | --- | --- | --- | --- | --- | --- | --- | --- | --- | --- | --- | --- | --- | --- | --- | --- | --- | --- | --- | --- | --- | --- | --- | --- | --- | --- | --- | --- | --- | --- | --- | --- | --- | --- | --- | --- | --- | --- | --- | --- | --- | --- | --- | --- | --- | --- | --- | --- | --- | --- | --- | --- | --- | --- | --- | --- | --- | --- | --- | --- | --- | --- | --- | --- | --- | --- | --- | --- | --- | --- | --- | --- | --- | --- | --- | --- | --- | --- | --- | --- | --- | --- | --- | --- | --- | --- | --- | --- | --- | --- | --- | --- | --- | --- | --- | --- | --- | --- | --- | --- | --- | --- | --- | --- | --- | --- | --- | --- | --- | --- | --- | --- | --- | --- | --- | --- | --- | --- | --- | --- | --- | --- | --- | --- | --- | --- | --- | --- | --- | --- | --- | --- | --- | --- | --- | --- | --- | --- | --- | --- | --- | --- | --- | --- | --- | --- | --- | --- | --- | --- | --- | --- | --- | --- | --- | --- | --- | --- | --- | --- | --- | --- | --- | --- | --- | --- | --- | --- | --- | --- | --- | --- | --- | --- | --- | --- | --- | --- | --- | --- | --- | --- | --- | --- | --- | --- | --- | --- | --- | --- | --- | --- | --- | --- | --- | --- | --- | --- | --- | --- | --- | --- | --- | --- | --- | --- | --- | --- | --- | --- | --- | --- | --- | --- | --- | --- | --- | --- | --- | --- | --- | --- | --- | --- | --- | --- | --- | --- | --- | --- | --- | --- | --- | --- | --- | --- | --- | --- | --- | --- | --- | --- | --- | --- | --- | --- | --- | --- | --- | --- | --- | --- | --- | --- | --- | --- | --- | --- | --- | --- | --- | --- | --- | --- | --- | --- | --- | --- | --- | --- | --- | --- | --- | --- | --- | --- | --- | --- | --- | --- | --- | --- | --- | --- | --- | --- | --- | --- | --- | --- | --- | --- | --- | --- | --- | --- | --- | --- | --- | --- | --- | --- | --- | --- | --- | --- | --- | --- | --- | --- | --- | --- | --- | --- | --- | --- | --- | --- | --- | --- | --- | --- | --- | --- | --- | --- | --- | --- | --- | --- | --- | --- | --- | --- | --- | --- | --- | --- | --- | --- | --- | --- | --- | --- | --- | --- | --- | --- | --- | --- | --- | --- | --- | --- | --- | --- | --- | --- | --- | --- | --- | --- | --- | --- | --- | --- | --- | --- | --- | --- | --- | --- | --- | --- | --- | --- | --- | --- | --- | --- | --- | --- | --- | --- | --- | --- | --- | --- | --- | --- | --- | --- | --- | --- | --- | --- | --- | --- | --- | --- | --- | --- | --- | --- | --- | --- | --- | --- | --- | --- | --- | --- | --- | --- | --- | --- | --- | --- | --- | --- | --- | --- | --- | --- | --- | --- | --- | --- | --- | --- | --- | --- | --- | --- | --- | --- | --- | --- | --- | --- | --- | --- | --- | --- | --- | --- | --- | --- | --- | --- | --- | --- | --- | --- | --- | --- | --- | --- | --- | --- | --- | --- | --- | --- | --- | --- | --- | --- | --- | --- | --- | --- | --- | --- | --- | --- | --- | --- | --- | --- | --- | --- | --- | --- | --- | --- | --- | --- | --- | --- | --- | --- | --- | --- | --- | --- | --- | --- | --- | --- | --- | --- | --- | --- | --- | --- | --- | --- | --- | --- | --- | --- | --- | --- | --- | --- | --- | --- | --- | --- | --- | --- | --- | --- | --- | --- | --- | --- | --- | --- | --- | --- | --- | --- | --- | --- | --- | --- | --- | --- | --- | --- | --- | --- | --- | --- | --- | --- | --- | --- | --- | --- | --- | --- | --- | --- | --- | --- | --- | --- | --- | --- | --- | --- | --- | --- | --- | --- | --- | --- | --- | --- | --- | --- | --- | --- | --- | --- | --- | --- | --- | --- | --- | --- | --- | --- | --- | --- | --- | --- | --- | --- | --- | --- | --- | --- | --- | --- | --- | --- | --- | --- | --- | --- | --- | --- | --- | --- | --- | --- | --- | --- | --- | --- | --- | --- | --- | --- | --- | --- | --- | --- | --- | --- | --- | --- | --- | --- | --- | --- | --- | --- | --- | --- | --- | --- | --- | --- | --- | --- | --- | --- | --- | --- | --- | --- | --- | --- | --- | --- | --- | --- | --- | --- | --- | --- | --- | --- | --- | --- | --- | --- | --- | --- | --- | --- | --- | --- | --- | --- | --- | --- | --- | --- | --- | --- | --- | --- | --- | --- | --- | --- | --- | --- | --- | --- | --- | --- | --- | --- | --- | --- | --- | --- | --- | --- | --- | --- | --- | --- | --- | --- | --- | --- | --- | --- | --- | --- | --- | --- | --- | --- | --- | --- | --- | --- | --- | --- | --- | --- | --- | --- | --- | --- | --- | --- | --- | --- | --- | --- | --- | --- | --- | --- | --- | --- | --- | --- | --- | --- | --- | --- | --- | --- | --- | --- | --- | --- | --- | --- | --- | --- | --- | --- | --- | --- | --- | --- | --- | --- | --- | --- | --- | --- | --- | --- | --- | --- | --- | --- | --- | --- | --- | --- | --- | --- | --- | --- | --- | --- | --- | --- | --- | --- | --- | --- | --- | --- | --- | --- | --- | --- | --- | --- | --- | --- | --- | --- | --- | --- | --- | --- | --- | --- | --- | --- | --- | --- | --- | --- | --- | --- | --- | --- | --- | --- | --- | --- | --- | --- | --- | --- | --- | --- | --- | --- | --- | --- | --- | --- | --- | --- | --- | --- | --- | --- | --- | --- | --- | --- | --- | --- | --- | --- | --- | --- | --- | --- | --- | --- | --- | --- | --- | --- | --- | --- | --- | --- | --- | --- | --- | --- | --- | --- | --- | --- | --- | --- | --- | --- | --- | --- | --- | --- | --- | --- | --- | --- | --- | --- | --- | --- | --- | --- | --- | --- | --- | --- | --- | --- | --- | --- | --- | --- | --- | --- | --- | --- | --- | --- | --- | --- | --- | --- | --- | --- | --- | --- | --- | --- | --- | --- | --- | --- | --- | --- | --- | --- | --- | --- | --- | --- | --- | --- | --- | --- | --- | --- | --- | --- | --- | --- | --- | --- | --- | --- | --- | --- | --- | --- | --- | --- | --- | --- | --- | --- | --- | --- | --- | --- | --- | --- | --- | --- | --- | --- | --- | --- | --- | --- | --- | --- | --- | --- | --- | --- | --- | --- | --- | --- | --- | --- | --- | --- | --- | --- | --- | --- | --- | --- | --- | --- | --- | --- | --- | --- | --- | --- | --- | --- | --- | --- | --- | --- | --- | --- | --- | --- | --- | --- | --- | --- | --- | --- | --- | --- | --- | --- | --- | --- | --- | --- | --- | --- | --- | --- | --- | --- | --- | --- | --- | --- | --- | --- | --- | --- | --- | --- | --- | --- | --- | --- | --- | --- | --- | --- | --- | --- | --- | --- | --- | --- | --- | --- | --- | --- | --- | --- | --- | --- | --- | --- | --- | --- | --- | --- | --- | --- | --- | --- | --- | --- | --- | --- | --- | --- | --- | --- | --- | --- | --- | --- | --- | --- | --- | --- | --- | --- | --- | --- | --- | --- | --- | --- | --- | --- | --- | --- | --- | --- | --- | --- | --- | --- | --- | --- | --- | --- | --- | --- | --- | --- | --- | --- | --- | --- | --- | --- | --- | --- | --- | --- | --- | --- | --- | --- | --- | --- | --- | --- | --- | --- | --- | --- | --- | --- | --- | --- | --- | --- | --- | --- | --- | --- | --- | --- | --- | --- | --- | --- | --- | --- | --- | --- | --- | --- | --- | --- | --- | --- | --- | --- | --- | --- | --- | --- | --- | --- | --- | --- | --- | --- | --- | --- | --- | --- | --- | --- | --- | --- | --- | --- | --- | --- | --- | --- | --- | --- | --- | --- | --- | --- | --- | --- | --- | --- | --- | --- | --- | --- | --- | --- | --- | --- | --- | --- | --- | --- | --- | --- | --- | --- | --- | --- | --- | --- | --- | --- | --- | --- | --- | --- | --- | --- | --- | --- | --- | --- | --- | --- | --- | --- | --- | --- | --- | --- | --- | --- | --- | --- | --- | --- | --- | --- | --- | --- | --- | --- | --- | --- | --- | --- | --- | --- | --- | --- | --- | --- | --- | --- | --- | --- | --- | --- | --- | --- | --- | --- | --- | --- | --- | --- | --- | --- | --- | --- | --- | --- | --- | --- | --- | --- | --- | --- | --- | --- | --- | --- | --- | --- | --- | --- | --- | --- | --- | --- | --- | --- | --- | --- | --- | --- | --- | --- | --- | --- | --- | --- | --- | --- | --- | --- | --- | --- | --- | --- | --- | --- | --- | --- | --- | --- | --- | --- | --- | --- | --- | --- | --- | --- | --- | --- | --- | --- | --- | --- | --- | --- | --- | --- | --- | --- | --- | --- | --- | --- | --- | --- | --- | --- | --- | --- | --- | --- | --- | --- | --- | --- | --- | --- | --- | --- | --- | --- | --- | --- | --- | --- | --- | --- | --- | --- | --- | --- | --- | --- | --- | --- | --- | --- | --- | --- | --- | --- | --- | --- | --- | --- | --- | --- | --- | --- | --- | --- | --- | --- | --- | --- | --- | --- | --- | --- | --- | --- | --- | --- | --- | --- | --- | --- | --- | --- | --- | --- | --- | --- | --- | --- | --- | --- | --- | --- | --- | --- | --- | --- | --- | --- | --- | --- | --- | --- | --- | --- | --- | --- | --- | --- | --- | --- | --- | --- | --- | --- | --- | --- | --- | --- | --- | --- | --- | --- | --- | --- | --- | --- | --- | --- | --- | --- | --- | --- | --- | --- | --- | --- | --- | --- | --- | --- | --- | --- | --- | --- | --- | --- | --- | --- | --- | --- | --- | --- | --- | --- | --- | --- | --- | --- | --- | --- | --- | --- | --- | --- | --- | --- | --- | --- | --- | --- | --- | --- | --- | --- | --- | --- | --- | --- | --- | --- | --- | --- | --- | --- | --- | --- | --- | --- | --- | --- |
| **Binding sites** | **Forward primers** | **Reverse primers** |
| *1* | ATCAAGGCTACCACAGCCAG | GGGTGTCGAGAAGACCGAAG |
| *2* | CTCTCCAATTCGCTGACCCA | CCGGATCGGATAGGTGAAGC |
| *3* | GGAACCGTGCGAGGTCAG | CTAGCATACGCGCTTGCCG |
| *4-L* | AGGGGTTGGCAAAGATATCCT | TGGAACATAGCCACACCCAT |
| *4-R* | TTGCTCTTCTGGATTGCTGG | GGCGATGAGGGCAATATCAA |
| *5* | TGAAGTTGGAAGCTGGCCT | TTAGGGTCCATGTGCTCAGG |
| *6* | GAAGTGGGGCTCTGGGTATT | ATTTGCTAAGTTGGGCTGGC |
| *7* | TTAGTGTGCCCGGATGTCTT | CCATGGGGCATTTGGGAAAA |

**
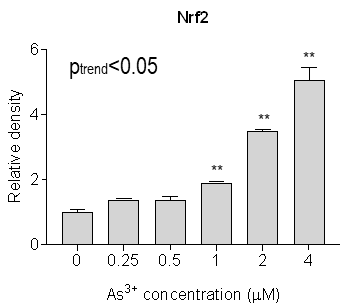
**

**Figure S1** Quantitative analysis of Nrf2 Western Blot from Figure 3A. Data are presented as mean ± SD (n = 3). **p* < 0.05 vs. the corresponding control, determined by one-way ANOVA with Bonferroni's multiple comparisons test.

**
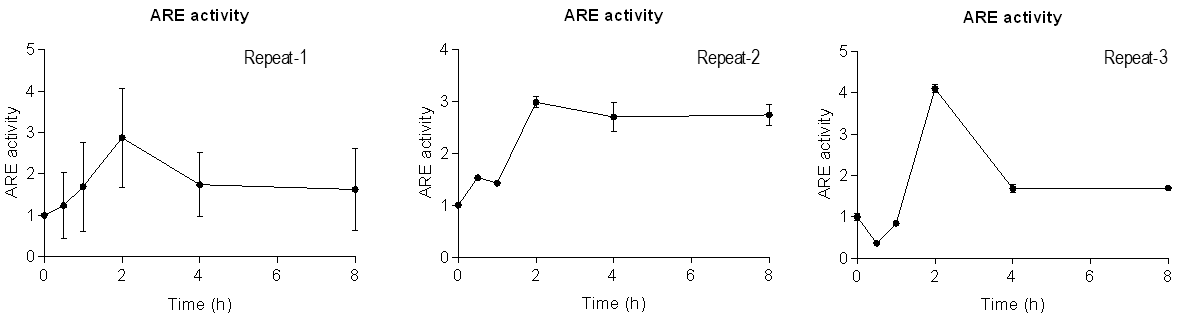
**

**Figure S2** ARE-Luciferase reporter assay for Nrf2 activation. ARE-inducible luciferase reporter assay showing Nrf2 activation over 0–8 h of treatment with 2 μM As³⁺. Data are presented as mean ± SD (n = 3). This experiment was repeated three times.

**
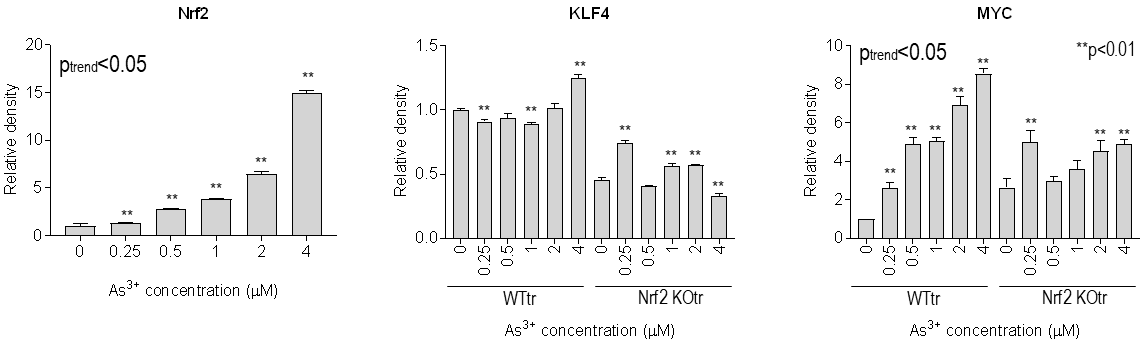
Figure S3** Quantitative Analysis of Nrf2, KLF4 and MYC Western Blot from Figure 5A. Data are presented as mean ± SD (n = 3). **p* < 0.05 vs. control, determined by one-way ANOVA with Bonferroni's multiple comparisons test.

**
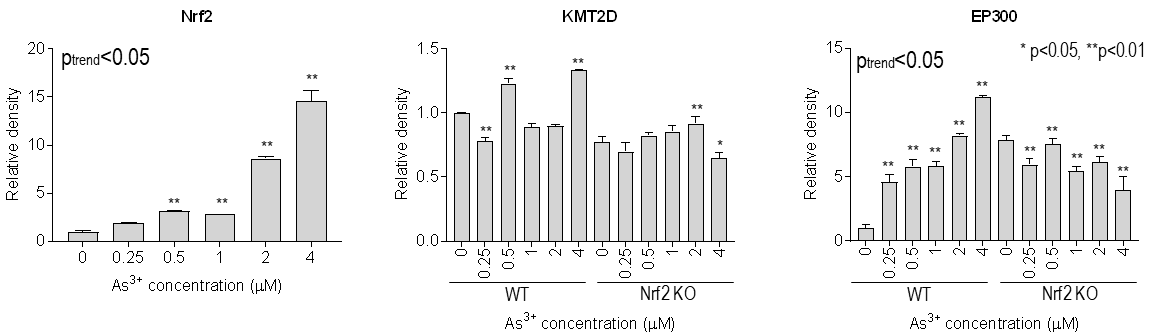
**

**Figure S4** Quantitative analysis of Nrf2, KMT2D and EP300 Western Blot from Figure 6B. Data are presented as mean ± SD (n = 3). **p* < 0.05 vs. the corresponding control, determined by one-way ANOVA with Bonferroni's multiple comparisons test.
